# Sequential‐Crosslinking Fibrin Glue for Rapid and Reinforced Hemostasis

**DOI:** 10.1002/advs.202308171

**Published:** 2023-12-10

**Authors:** Lisha Yu, Zhaodi Liu, Zongrui Tong, Yihang Ding, Zhefeng Qian, Weilin Wang, Zhengwei Mao, Yuan Ding

**Affiliations:** ^1^ Department of Hepatobiliary and Pancreatic Surgery The Second Affiliated Hospital School of Medicine Zhejiang University Hangzhou Zhejiang 310009 China; ^2^ Key Laboratory of Precision Diagnosis and Treatment for Hepatobiliary and Pancreatic Tumor of Zhejiang Province Hangzhou Zhejiang 310009 China; ^3^ Research Center of Diagnosis and Treatment Technology for Hepatocellular Carcinoma of Zhejiang Province Hangzhou Zhejiang 310009 China; ^4^ National Innovation Center for Fundamental Research on Cancer Medicine Hangzhou Zhejiang 310009 China; ^5^ Cancer Center Zhejiang University Hangzhou Zhejiang 310058 China; ^6^ ZJU‐Pujian Research & Development Center of Medical Artificial Intelligence for Hepatobiliary and Pancreatic Disease Hangzhou Zhejiang 310058 China; ^7^ MOE Key Laboratory of Macromolecular Synthesis and Functionalization Department of Polymer Science and Engineering Zhejiang University Hangzhou Zhejiang 310027 China

**Keywords:** biomaterials, fibrin glue, hemostasis, hydrogel, sequential‐crosslinking

## Abstract

Achieving hemostasis effectively is essential for surgical success and excellent patient outcomes. However, it is challenging to develop hemostatic adhesives that are fast‐acting, strongly adherent, long‐lasting, and biocompatible for treating hemorrhage. In this study, a sequential crosslinking fibrin glue (SCFG) is developed, of which the first network of the fibrin glue forms in situ within 2 s to act as an initial physical barrier and locks the gelatin methacryloyl precursor for tight construction of the second network to enhance wet adhesion and durability for tissues covered with blood. The sequential crosslinking glue can provide large pressures (≈280 mmHg of burst pressure), makes strong (38 kPa of shear strength) and tough (≈60 J m^−2^ of interfacial toughness) interfaces with wet tissues, and outperforms commercial hemostatic agents and gelatin methacryloyl. SCFG are demonstrated as an effective and safe sealant to enhance the treatment outcomes of bleeding tissues in rat, rabbit, and pig models. The ultrafast gelation, strong adhesion and durability, excellent compatibility, and easy manufacture of SCFG make it a promising hemostatic adhesive for clinical applications.

## Graphical abstract

A sequential crosslinking fibrin glue (SCFG), of which the initial network of the fibrin glue forms within 2 s and locks the gelatin methacryloyl precursor for construction of the second network to enhance wet adhesion and durability for tissues, is developed as a promising hemostatic adhesive.

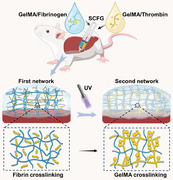

## 1 Introduction

Uncontrolled hemorrhage from trauma is the main cause of global mortality, which accounts for more than 2 million deaths annually.^[^ ^1^, ^2^, ^3^ ^]^ Hemorrhage management is time sensitive; therefore, rapid hemostatic control can promote the survival of injured patients.^[^ ^4^, ^5^, ^6^ ^]^ Existing topical hemostatic agents depend on speeding up blood coagulation,^[^ ^4^, ^7^, ^8^ ^]^ which is accomplished either by the local administration of pro‐coagulants^[^ ^9^ ^]^ or by concentrating coagulation components via fast water absorption.^[^ ^10^, ^11^ ^]^ However, a weak blood clot formed in the wound bed can be washed away by rapid or pressured blood flow, limiting the efficiency of hemostasis.^[^ ^12^ ^]^ Hemostatic technologies and devices that only use blood coagulation cannot provide immediate bleeding control.^[^ ^13^ ^]^

The adhesive sealing of bleeding tissues is a promising alternative for surgical hemostasis.^[^ ^12^, ^13^, ^14^ ^]^ Notably, several wet tissue adhesives have been developed to improve adhesion performance.^[^ ^15^, ^16^, ^17^, ^18^, ^19^ ^]^ These tissue adhesives function through two major mechanisms^[^ ^20^, ^21^, ^22^ ^]^: i) interfacing with the chemistry of tissues, and ii) maintaining adhesion with physical characteristics. However, adhesives that can create strong chemical and mechanical interactions with wet tissue^[^ ^16^, ^23^, ^24^, ^25^ ^]^ unavoidably suffer from severe dilution and swelling upon rapid and pressured blood flow.^[^ ^13^ ^]^ This effect degrades the mechanical performance and largely compromises the stability of tissue adhesives against the loss of components. Meanwhile, their utility for hemostasis is limited by the requirement for prolonged gelation time and interfacial reaction,^[^ ^15^, ^24^ ^]^ as well as the application of pressure to form strong adhesion.^[^ ^12^, ^13^, ^26^ ^]^

The ideal design of a bleeding tissue adhesive should deliver fast, strong adhesion, be long‐lasting, and provide biocompatible treatment for the hemorrhage.^[^ ^27^, ^28^ ^]^ This design requires^[^ ^22^, ^29^ ^]^ (i) quick gelation, enabling immediate blood control and leak prevention; (ii) strong adhesion that allows long‐lasting application; and (iii) a nontoxic and biocompatible hemostatic system that is easy to operate. In 1998, fibrin glue was approved as a tissue adhesive that contains fibrinogen and thrombin.^[^ ^30^, ^31^ ^]^ Fibrin glue mimics the gradual nature of blood clot formation and exhibits ultrafast gelation,^[^ ^20^, ^21^ ^]^ both of which are crucial features for providing a physical barrier and slowing the blood flow. Inherently weak adhesion and mechanical performance limit the hemostatic effect.^[^ ^21^ ^]^ Common strategies that rely on adding excipients such as collagen, gelatin, albumin, and chitin for improving mechanical properties are still limited by weak adhesion to wet tissue and rapidly pressurized blood.^[^ ^4^, ^32^, ^33^, ^34^ ^]^ Molecular entanglement and energy dissipation can contribute to the remarkable mechanical performance of double‐network hydrogels.^[^ ^20^, ^35^, ^36^ ^]^ The introduction of a second network into the fibrin glue can further enhance the shear strength and adhesive energy, which results in a stronger physical barrier for pressurized blood. Gelatin is a derivative of collagen with good compatibility and low cost and can be modified to have functional groups such as methacrylate (gelatin methacryloyl, GelMA).^[^ ^37^, ^38^ ^]^ Photoinduced crosslinking adhesives have attracted considerable interest because of their controllable gelation time and tunable physical properties.^[^ ^39^, ^40^, ^41^ ^]^ GelMA can be employed as a secondary network for ensuring strong adhesion between adhesives and tissues.^[^ ^23^, ^42^, ^43^ ^]^ A simple combination of fibrin glue and a second polymer network can provide a fast‐gelation hemostatic adhesive with desirable mechanical properties for clinical applications.

In this paper, we report on a sequential crosslinking fibrin glue (SCFG), of which the first network of fibrin glue forms in situ within 2 s to act as an initial physical barrier and locks the GelMA precursor for tight construction of the second network to enhance wet adhesion and durability for tissues covered with blood. The sequential crosslinking glue can provide stronger adhesion than fibrin glue in terms of shear strength, interfacial toughness, and adhesive strength tests. We further demonstrate SCFG as an effective sealant to enhance the treatment outcomes of bleeding liver and heart tissues in rat, rabbit, and pig models.

## 2 Results

### 2.1 Preparation and Characterizations of SCFG

**Figure 1:**
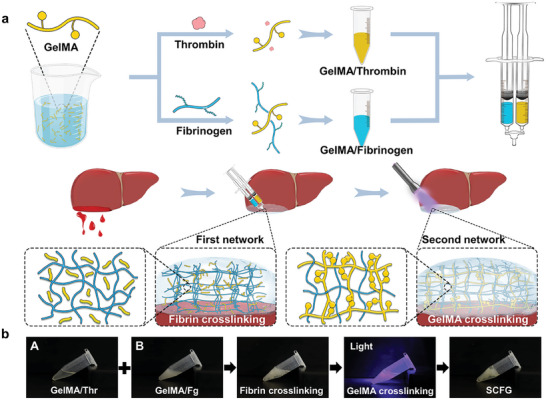
Preparation and characterizations of SCFG. a), Schematic of preparation and application of SCFG. b), Digital photograph of SCFG formation process. Double networks are formed in two steps.

The proposed strategy used SCFG by combining the ultrafast gelation of fibrin glue and the strong adhesion of GelMA to achieve rapid and tough hemostatic sealing. The preparation process for SCFG is simple and involves mixing the two components of fibrin glue with GelMA. The hemostatic bioadhesive is used as an injectable solution that comprises a precursor solution with photo‐initiating GelMA and clottable fibrinogen (GelMA/Fg) and another precursor solution with photo‐initiating GelMA and thrombin (GelMA/Thr) (**Figure** **1**a). The fast gelation of fibrin glue helps reduce the loss of uncross‐linked polymers and promotes the retention of hydrogels in a fluidically and mechanically dynamic environment.^[^ ^44^ ^]^ When these two types of injectable solutions were mixed, the first network was immediately formed via thrombin‐mediated fibrinogen‐to‐fibrin mesh conversion, and the second network was subsequently cross‐linked with light‐induced GelMA polymerization (Figure 1b; Movie S1, Supporting Information). At the bleeding injury site, the first network of SCFG (fibrin mesh) provides an initial physical barrier and prevents loss of the GelMA precursor solution at the wound site. Then, the second network of GelMA solidifies rapidly in situ and protects the fibrin clot from being washed away, strengthening the adhesion to seal the wound. SCFG exhibits a short gelation time and strong adhesion properties, which ensures rapid and robust hemostatic sealing.

The composition ratios of the mechanical properties of SCFG were studied using rheological tests (Figure S1, Supporting Information). The mechanical performance of SCFG improved with increasing concentration of fibrinogen and GelMA up to 5%(w/v) and 9% (w/v), respectively. Further increases in fibrinogen and GelMA concentrations did not enhance mechanical performance. Therefore, the composition concentration ((fibrinogen, 5%(w/v); GelMA, 9% (w/v)) was utilized to assess both the mechanical performance and hemostatic efficiency. Rheological tests further showed that the storage modulus of SCFG (2790 ± 207 Pa) was considerably higher than that of the fibrin mesh (245 ± 13 Pa) and GelMA hydrogel (1270 ± 332 Pa) (Figure S2, Supporting Information). The enhanced mechanical properties of SCFG are important for resisting blood pressure during hemostasis. Further, the rapid gelation of the as‐synthesized SCFG was perfectly inherited from the pristine fibrin glue with a gelation time of 1.3 ± 0.3 s, and the presence of blood does not affect the gelation time (Figures S3 and S4, Supporting Information). The stored precursor solution exhibited rapid gelation and maintained excellent mechanical performance for up to 6 h at room temperature, meeting the requirements of most surgical procedures (Figures S5 and S6, Supporting Information). We believe that this two‐step sequential crosslinking of hybrid fibrin glue can achieve ultrafast gelation, which makes SCFG ideal for mitigating challenging clinical conditions.

The crosslinking networks and swelling properties of hydrogels play major roles in their mechanical performance.^[^ ^45^ ^]^ Field‐emission scanning electron microscopy (FE‐SEM) and confocal laser scanning microscope (CLSM) images confirmed that the SCFG structure was derived from both the fibrin mesh and the GelMA network (Figure S7, Supporting Information). The two types of networks were dispersed homogeneously and interspersed. Then, we compared the mechanical properties of SCFG to those of fibrin glue and GelMA when used alone. The stretchability, tensile strength, and fracture energy of SCFG were measured in comparison to fibrin glue and GelMA independently (Figure S8, Supporting Information). The fracture energy of SCFG (12.12 ± 0.32 J m^−2^) was higher than that of fibrin glue (3.01 ± 1.02 J m^−2^) and GelMA (6.62 ± 1.89 J m^−2^). The better tensile strength, and fracture energy of SCFG validated the benefits of combining these two materials. Further, this was verified by the swelling ratio test,^[^ ^46^ ^]^ which suggests that the degree of crosslinking in SCFG was higher than that in the fibrin glue and GelMA (Figure S9, Supporting Information). The reduced swelling ratio of SCFG is beneficial for sealant applications because of its minimal deformation and strong interaction with wet tissues.^[^ ^46^ ^]^ We believe that a homogeneously interspersed hydrogel can provide sufficient hemostatic sealing.

### 2.2 Adhesion Performance of SCFG

**Figure 2:**
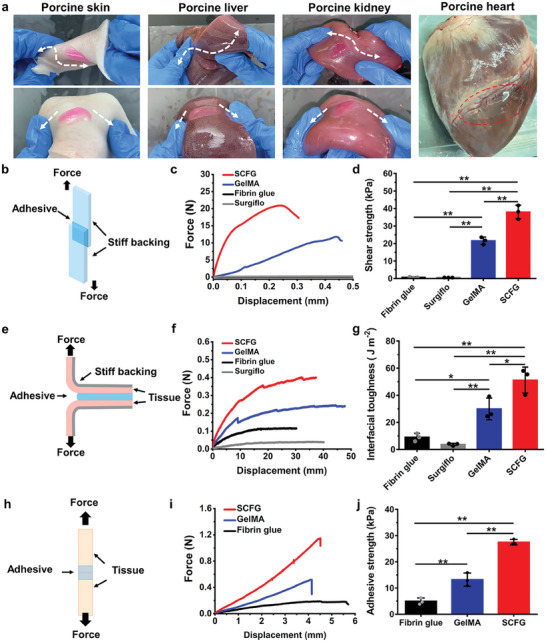
Adhesion performance of SCFG. a), Photograph of SCFG on porcine tissues. b), Schematic of the lap shear test used for determining shear strength. c), Force–displacement curves of the Lap‐shear test. d), Lap shear strength of fibrin glue, GelMA, Surgiflo, SCFG. e), Schematic of T‐peel adhesion test. f), Force–displacement curves of the T‐peel adhesion test. g), Interfacial toughness values of fibrin glue, GelMA, Surgiflo, and SCFG. h), Schematic of the end‐to‐end adhesive strength. i), Force–displacement curves of adhesive strength. j), Adhesive strengths of fibrin glue, GelMA, and SCFG. P values are determined by one‐way ANOVA followed by the Tukey's comparison test. Error bars, mean ± SD. ^*^ *p* < 0.05; ^**^ *p* < 0.01.

The adhesion performance of a hemostatic hydrogel is crucial for sealing an injured site.^[^ ^38^ ^]^ The ability of SCFG to adhere to wet tissues was evaluated in vitro to demonstrate the potential applications. When SCFG was injected into porcine tissues via a two‐step sequential crosslinking process, it tightly adhered to fresh tissues (porcine skin, liver, kidney, and heart) and withstood twisting and bending operations (**Figure** **2**a).

Given the dynamics of biological tissues and organs, both the longitudinal shear stress and transverse tensile stress at the wound site can cause lacerations and blood leakages.^[^ ^47^ ^]^ Therefore, the adhesion performance of SCFG was evaluated for shear strength, interfacial toughness, and adhesive strength based on the American Society for Testing Material (ASTM) standards. For comparison, we included GelMA hydrogel, commercial fibrin glue, and Surgiflo (flowable gelatin matrix) as control groups (Table S1, Supporting Information); the results indicate that SCFG exhibited superior adhesion properties in all three tests. The shear strength of SCFG (38.00 ± 3.90 kPa) was considerably higher than that of the GelMA hydrogel (21.60 ± 2.11 kPa) and fibrin glue (0.69 ± 0.29 kPa) because of the homogeneously interspersed hydrogels of the fibrin mesh and GelMA (Figure 2b–d). In the T‐peel adhesion test, the interface toughness reached 51.20 ± 9.61 J m^−2^ for SCFG, which was higher than that of GelMA (26.82 ± 11.99 J m^−2^), fibrin glue (8.70 ± 2.45 J m^−2^) and Surgiflo (3.74 ± 0.85 J m^−2^), as showed in Figure 2e–g. Commercial fibrin glue and Surgiflo exhibited poor adhesion performance, rendering them unsuitable for severe hemorrhage. Furthermore, adhesive strength was investigated to evaluate wound closure capacity. SCFG (27.50 ± 1.04 kPa) consistently presented a stronger adhesive strength than that of GelMA hydrogel (13.23 ± 2.52 kPa) and fibrin glue (4.98 ± 1.26 kPa). The combination of high mechanical properties and adhesion performance allows SCFG to seal the flows of body fluids. SCFG exhibited high burst pressure at 278.3 ± 22.1 mmHg, in contrast to the weak sealing of fibrin glue and GelMA (Figure S10, Supporting Information). Various widely used biopolymer gels were also incorporated as the second crosslinking networks in the formation of SCFG, significantly enhancing the adhesion performance without affecting the gelation time (Figures S11–S14, Supporting Information). The excellent adhesion and sealing performance can effectively prevent blood leakage, indicating its potential for sealing wet tissues in clinical applications.

### 2.3 Biocompatibility and Biodegradation of SCFG

**Figure 3:**
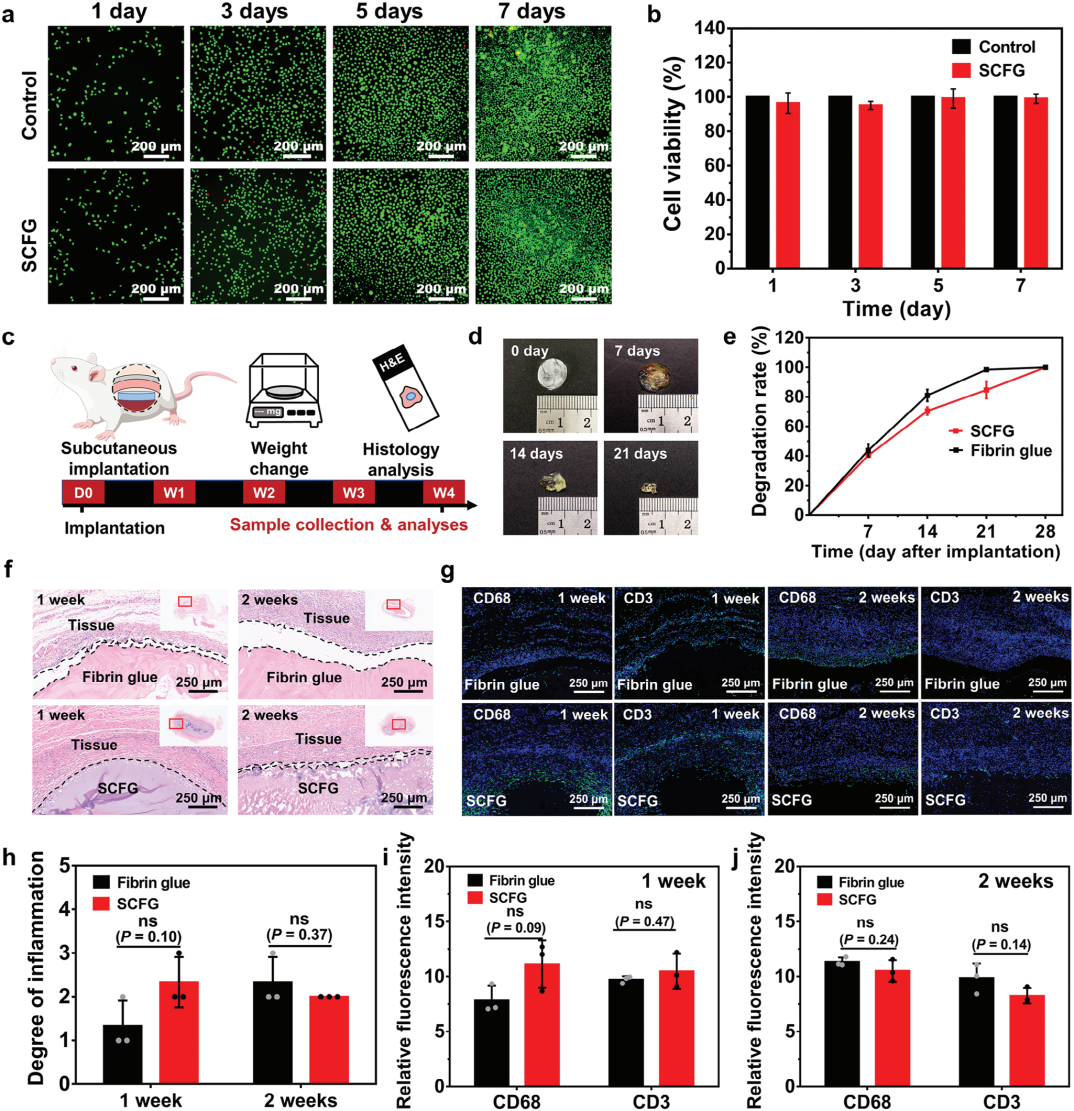
Biocompatibility and biodegradability of SCFG. a), Live–dead staining of L929 fibroblasts in control media and SCFG‐incubated media for 1, 3, 5, and 7 days; L929 fibroblasts were stained with calcein acetoxymethyl to detect living cells (green) and propidium iodide to detect dead cells (red). b), Cytotoxicity of SCFG to L929 fibroblasts after incubation for 1, 3, 5, and 7 days (*n* = 3 per group). c), Schematic of dorsal subcutaneous implantation in rat models and sample analyses. d), Image of the subcutaneously implanted SCFG at 0, 7, 14, and 21 days after implantation. e), Degradation rate of subcutaneously implanted SCFG and fibrin glue (*n* = 3). f), Representative histology images stained with hematoxylin and eosin (H&E) for fibrin glue (top) and SCFG (bottom) with the surrounding tissue at 1 and 2 weeks after subcutaneous implantation. g), Representative immunofluorescence images for fibrin glue and SCFG at 1 and 2 weeks. Blue fluorescence corresponds to cell nuclei stained with 4′,6‐diamidino‐2‐phenylindole (DAPI); green fluorescence corresponds to the expression of pan‐macrophage (CD68) and T cells (CD3). h), Degree of inflammation from histology images evaluated by a blinded pathologist (0, normal; 1, very mild; 2, mild; 3, severe; 4, very severe). Relative fluorescence intensity from the immunofluorescence images for pan‐macrophage (CD68) and T cells (CD3) in fibrin glue and SCFG groups at i) 1 and j) 2 weeks after subcutaneous implantation (*n* = 3). P values are determined by a two‐sided *t*‐test. Error bars, mean ± SD. ns. not significant, ^*^ *p* < 0.05; ^**^ *p* < 0.01.

We performed in vitro and in vivo characterizations using rat models to evaluate the biocompatibility and biodegradability of SCFG (**Figure** **3**). The in vitro cytotoxicity of SCFG was evaluated using CCK‐8 and live/dead assays. Compared to the control, SCFG had a comparable effect on cell viability and growth after treatment for 1, 3, 5, and 7 days (Figure 3a,b; Figure S15, Supporting Information), indicating its excellent cell compatibility and potential safety for further applications.

We evaluated the in vivo biodegradability and biocompatibility of SCFG by dorsal subcutaneous implantation in a rat model followed by assessment at various time points from 1–4 weeks (Figure 3c). Fibrin glue was implanted in the control group. Both SCFG and fibrin glue exhibited a gradual decrease in weight during the implantation period, with a substantial degradation observed 4 weeks after implantation (Figure 3d,e). An appropriate sealant degradation rate is critical to ensure that the sealant material does not completely degrade before tissue healing.^[^ ^48^ ^]^ The histological evaluation results demonstrated that SCFG induced mild inflammation in the surrounding tissues compared with that using the fibrin glue (Figure 3f,h). We conducted an evaluation of the biocompatibility of SCFG through immunofluorescence staining and relative fluorescence intensity analysis of common markers associated with inflammation and foreign body response (Figure 3g,i,j; Figure S16, Supporting Information). These markers include macrophages (CD68 for pan‐macrophage, CD206 for M2 type) and T cells (CD3). The immunofluorescence images and the corresponding relative fluorescence intensity of these biomarkers revealed that SCFG induced a similar level of inflammatory and foreign body response when compared to fibrin glue. SCFG has excellent biocompatibility and biodegradability, which is a promising candidate for clinical applications.

### 2.4 Rapid and Tough Hemostatic Sealing

The rapid and robust adhesion of SCFG to wet tissues has considerable potential for application to hemostatic sealing. The hemostatic capacity of SCFG was evaluated in rat, rabbit, and porcine injury models.

**Figure 4:**
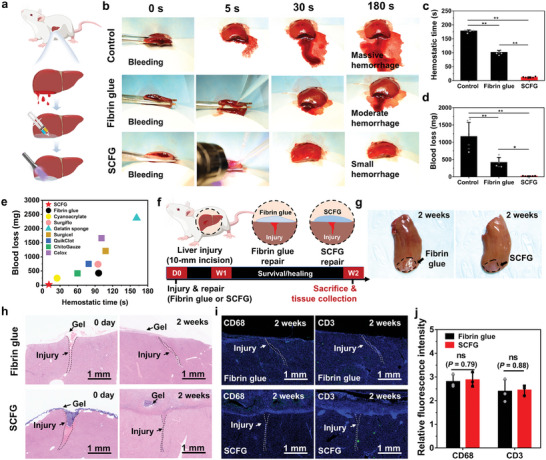
In vivo hemostatic sealing in a rat model. a), Schematic of liver bleeding in the rat model. A part of the liver lobe was removed (length:3 cm, width:0.5 cm) to establish a massive bleeding model. b), Experimental images of the hemostatic treatment of liver defect in the blank control, fibrin glue, and SCFG groups. c) Hemostatic time and d) blood loss of the rat model with a severe liver injury (*n* = 6). e), Comparison of the hemostatic performance of the SCFG with that of commercial hemostatic materials (*n* = 3). f), Schematic of implanting the liver injury in the rat model. g), Rat liver at 2 weeks after sealing with fibrin glue and SCFG, respectively. h), Representative histology images stained with H&E for fibrin glue and SCFG at 0 days and 2 weeks after hemostatic treatment. i), Representative immunofluorescence images for fibrin glue and SCFG at 2 weeks after hemostatic treatment. Blue fluorescence corresponds to cell nuclei stained with 4′,6‐diamidino‐2‐phenylindole (DAPI); green fluorescence corresponds to the expression of pan‐macrophage (CD68) and T cells (CD3). j), Relative fluorescence intensity from the immunofluorescence images for CD68 and CD3 (*n* = 3). P values are determined by a two‐sided *t*‐test for the comparisons between two groups and by one‐way ANOVA followed by the Tukey's comparison test for the comparison between multiple groups. Error bars, mean ± SD. ns. not significant, ^*^ *p* < 0.05; ^**^ *p* < 0.01.

The hemostatic sealing capability of SCFG was quantitatively assessed via the severe liver injury rat model, which is a well‐established model for determining hemostatic capability (**Figure** **4**a).^[^ ^38^, ^47^ ^]^ Part of the liver lobe (length, 3 cm; width, 0.5 cm) was removed to establish a massive bleeding model. Hemostatic treatment was applied to the sections using fibrin glue and SCFG. The fibrin glue has characteristics similar to those of soft tissues; however, it provides low adhesion, particularly to wet tissues. When commercial fibrin glue was applied to the injured site, blood flowed from the wound (Figure 4b; Movie S2, Supporting Information). SCFG exhibited rapid and strong adhesion to the injured site and achieved hemostatic sealing within 15 s (Figure 4b; Movie S3, Supporting Information). Thus, SCFG exhibited a considerably shorter hemostatic time than that of fibrin glue (101 ± 7 s, Figure 4c). Blood loss in the SCFG group (20 ± 8 mg) was ≈95% less than that in the fibrin glue group (415 ± 145 mg, Figure 4d). To augment the hemostatic performance of GelMA‐based adhesives, procoagulant materials are incorporated into the hydrogel, or alternatively, second networks are utilized for crosslinking with GelMA (Table S2, Supporting Information).^[^ ^23^, ^38^ ^]^ A hemostatic adhesive consisting of GelMA and hemocoagulase, a thrombin‐like proteinase that catalyzes the conversion of fibrinogen to fibrin, accelerates fibrin formation and creates a physical barrier that prevents bleeding.^[^ ^38^ ^]^ In the severe liver injury rat model, GelMA and GelMA/Thr (GelMA and thrombin) were prepared as reference samples (Figure S17, Supporting Information). Compared with GelMA and GelMA/Thr, SCFG exhibited better hemostatic performance in terms of shorter hemostatic time and low blood loss. When added to the bleeding injury site, SCFG immediately formed the first network of fibrin mesh and promoted strong adhesion of GelMA polymerization. The outstanding hemostatic capability of SCFG has been corroborated in instances of acute hemorrhage, including a rat heart bleeding model^[^ ^12^, ^49^ ^]^ (Figure S18, Supporting Information), as well as in scenarios of massive hemorrhage in rabbit liver penetration and hepatectomy models (Figure S19, Supporting Information).

Existing commercial hemostatic materials, which include adhesives (cyanoacrylate), hemostatic matrices (Surgiflo), hemostatic sponges (gelatin sponge), and hemostatic gauzes (Surgicel, Quikclot, ChitoGauze, and Celox), were also used as reference groups (Table S1, Supporting Information). A critical limitation of most commercially available materials is their low adhesive strength, particularly in biological and dynamic environments. Some topical hemostatic agents (Surgicel and gelatin sponge) can speed up blood coagulation but exhibit low efficiency for acute hemorrhage. Cyanoacrylate‐based sealants exhibit high adhesive strength but are more rigid than native tissues and have high cytotoxicity and inflammatory responses.^[^ ^50^, ^51^ ^]^ The major challenges of commercial hemostats include providing fast gelation and strong tissue adhesion with minimal toxicity, host inflammation, and side effects. Figure 4e illustrates that SCFG characterized by rapid gelation and robust tissue adhesion had a shorter hemostatic time and reduced blood loss compared to the commercial hemostatic materials.

The long‐term sealing and biocompatibility of SCFG were evaluated by examining SCFG 2 weeks after hemostasis in a rat liver injury model (Figure 4f). The SCFG maintained a seal on the injured rat liver model in vivo 2 weeks after the initial hemostatic application (Figure 4g). Histological analysis of the sealed rat liver tissues indicated that SCFG enabled cells to infiltrate into the crosslinked bioadhesive hydrogel and promoted the healing of underlying injuries (Figure 4h). Immunofluorescence analysis of biomarkers CD68 for pan‐macrophages and CD3 for T cells revealed that SCFG and fibrin glue induced similar levels of inflammation (Figure 4i,j). Owing to its biodegradable nature and strong adhesion, SCFG can accelerate the healing and regeneration of injured tissue. The promising biocompatibility and long‐term sealing capability of the SCFG make it a potential candidate for surgical applications.

**Figure 5:**
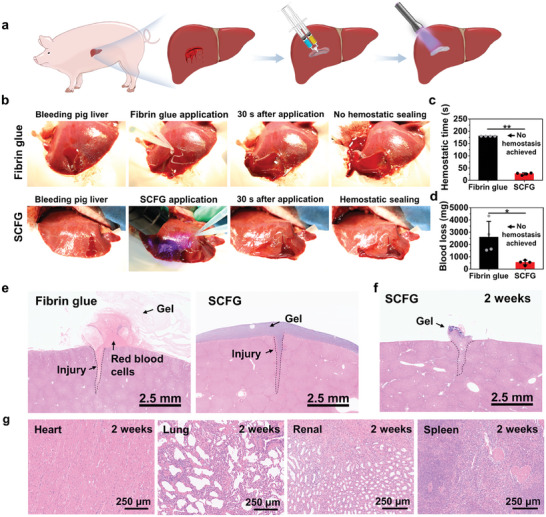
In vivo hemostatic sealing in a porcine liver bleeding model. a), Schematic of the establishment of the porcine liver bleeding model. An incision (length:1.5 cm, depth:0.3 cm) was made on the liver to establish the porcine liver bleeding model. b), Experimental images of the hemostatic treatment of liver defects in the fibrin glue and SCFG groups. c) Hemostatic time and d) blood loss in the porcine liver bleeding model (*n* = 4). e), H&E for the porcine liver defects treated with the fibrin glue and SCFG 0 days after hemostasis. f), H&E for porcine liver defects treated with the SCFG at 2 weeks after hemostasis. g), Representative histology images of porcine heart, lung, renal, and spleen stained with H&E at 2 weeks after hemostatic sealing by SCFG. P values are determined by a two‐sided *t*‐test. Error bars, mean ± SD. ns. not significant, ^*^ *p* < 0.05; ^**^ *p* < 0.01.

We demonstrated the rapid hemostatic sealing of bleeding liver injuries in pigs to further validate the in vivo efficacy of SCFG in a more clinically relevant setting. An incision (length:1.5 cm; depth:0.3 cm) was created on the liver to establish the porcine liver bleeding model (**Figure** **5**a). To provide hemostatic sealing, fibrin glue was injected into the bleeding site. The hemostatic seal between the fibrin glue and the liver surface failed because of the persistent brisk bleeding and hard‐to‐reach bleeding tissue surfaces (Figure 5b). The injured site injected with fibrin glue was observed via H&E staining, which confirmed that the fibrin glue was easily washed away by the blood flow (Figure 5e). After 3 min of application, the fibrin glue failed to achieve hemostatic sealing (Figure 5c). In contrast, persistent brisk bleeding decreased rapidly when SCFG was injected into the wound site (Figure 5b,e). Finally, SCFG provided rapid and effective hemostatic sealing of bleeding porcine liver injuries within 30 s. The blood loss in the SCFG group (533 ± 187 mg) was ≈80% less than that in the fibrin glue group (2584 ± 1304 mg, Figure 5d). The SCFG instantly adhered to the liver, providing a protective barrier to seal the injured tissue for a long time.

After the hemostasis of the bleeding liver injury by SCFG, all the pigs survived. The animals did not show any signs of adverse reactions during the 2‐week observation period. The histological analysis of the sealed liver tissues 2 weeks after hemostatic application showed that SCFG maintained a partial seal on the liver defect, enabling cell infiltration and injury healing (Figure 5f). The H&E staining of the main organs in the SCFG group revealed no apparent pathological changes (Figure 5g). Overall, we demonstrated that SCFG can effectively achieve rapid and robust hemostasis in severe trauma cases and possess excellent biocompatibility and biodegradability.

## 3 Conclusion

In summary, we developed a sequential crosslinking fibrin glue (SCFG), of which the first network of the fibrin glue forms in situ within 2 s to act as an initial physical barrier and locks the gelatin methacryloyl precursor for tight construction of the second network to enhance wet adhesion and durability for wet tissues. The sequential crosslinking glue can exhibit a synergistic effect and provide stronger adhesion than commercial fibrin glue in adhesion performance. We further demonstrated that SCFG is an effective and safe sealant to enhance the treatment outcomes of bleeding liver and heart tissues in animal models. The ultrafast gelation, strong adhesion, excellent compatibility, and storage stability of SCFG make it a promising hemostatic adhesive for clinical applications.

## 4 Experimental Section

### Materials

GelMA (60% substitution, 100–200 kDa) and polymerization initiator lithium phenyl‐2,4,6 trimethylbenzoylphosphinate (LAP) were obtained from Suzhou Yongqinquan Intelligent Equipment Co., Ltd. (Suzhou, China). Sodium chloride and calcium chloride were purchased from Sinopharm Chemical Reagent Co., Ltd. Commercial fibrin glue (Human Fibrin Sealant Kit) was obtained from Shanghai RAAS Blood Products Co., LTD. (Shanghai, China). Fibrinogen and thrombin from the Human Fibrin Sealant Kit were used for the preparation of SCFG. The concentration of fibrinogen was 5% (w/v) in SCFG and commercial fibrin glue for performance evaluation.

### Preparation of SCFG

SCFG is a two‐component precursor solution comprising GelMA/Fibrinogen (GelMA/Fg) and GelMA/Thrombin (GelMA/Thr). The GelMA prepolymer solution was prepared by dissolving freeze‐dried GelMA and LAP powder in 0.9% NaCl solution at 50 °C. The precursor of GelMA/Thr solution was prepared by adding thrombin solution to the GelMA prepolymer solution, with a concentration of 13%(w/v) GelMA, 1000 IU mL^−1^ thrombin, and 0.25%(w/v) LAP. Similarly, the precursor of GelMA/Fg solution was prepared by adding fibrinogen solution to the GelMA prepolymer solution, with the concentration of 5%(w/v) GelMA, 5%(w/v) fibrinogen, and 0.125%(w/v) LAP. The specific GelMA concentration ratio plays a critical role in ensuring the uniform dispersion of precursors, facilitating the formation of two interconnected cross‐linking networks. The protein entanglement of macromolecules may influence the molecular recognition and catalytic efficiency of thrombin for fibrinogen. This specific GelMA concentration ratio can ensure the catalytic efficiency of thrombin to generate the fibrin network before the formation of a second network.

A UV flashlight at a wavelength of 405 nm with a power of 3 W was employed for photo‐crosslinking. In the characterization and hemostatic evaluation, the duration of UV exposure was 10 s. In the mechanical tests, the duration of UV exposure was 1 min, because the gelation of SCFG was sandwiched between two slides (glass slides, two pieces of sausage skin membranes).

### Characterization

The chemical structure of the as‐prepared sample was characterized by proton nuclear magnetic resonance (^1^H NMR). As‐prepared samples were dissolved in D_2_O, and the ^1^H NMR spectra were obtained using a Bruker AVANCE NEO 600‐MHz NMR Spectrometer. The chemical shifts were represented in ppm. The grafted methacrylate was characterized by ^1^H NMR by comparing the characteristic peaks of gelatin and GelMA.

Field‐emission scanning electron microscope (FE‐SEM) analysis was performed to evaluate the morphology of the samples. The samples were frozen and then dried under vacuum at −80 °C overnight. Samples were sputter‐coated with gold before analysis, and SEM images were obtained by using a Zeiss Sigma 300 SEM at 3 kV. To further assess the morphology of the SCFG, 3D images of the cross‐linked gel were obtained using confocal laser scanning microscopy (CLSM) (Zeiss LSM900). Cyanine 3 NHS‐ester was used to prepare the Cyanine 3‐labeled fibrinogen.^[^ ^52^ ^]^ FITC‐labeled GelMA was obtained from Suzhou Yongqinquan Intelligent Equipment Co., Ltd. (Suzhou, China). The FITC‐labeled GelMA/Thr precursor solution and the FITC‐labeled GelMA/Cy3‐labeled Fg precursor solution were injected in Petri dishes to form the cross‐linked SCFG. The samples were frozen and then dried under vacuum at −80 °C overnight. The 3D CLSM image was generated from z‐stacked x‐y slice images.

### Lap Shear Test

The lap shear test was performed according to a previous study,^[^ ^38^ ^]^ using the modified ASTM F2255‐05 standard. Briefly, 200 µL of the sample was injected onto the adhesion zone (20 × 25 mm) between the surfaces of two pieces of glass slides, and photo‐crosslinked by 405 nm light for 1 min. Two glass slides were then placed into an Instron mechanical tester (Zwick/Roell Z020 with a 1 kN sensor) with a strain rate of 4 mm min^−1^. The shear strength was determined at the point of detachment. All measurements were repeated three times.

### T‐Peel Adhesion Test

The T‐peel test was performed according to a previous study,^[^ ^16^ ^]^ using the modified ASTM F2256‐05 standard. The sausage skin membrane was bonded to a rigid polyethylene terephthalate (PET) film with Cyanoacrylate glue. One end of the PET film was kept open to limit deformation at the crack tip. Subsequently, 1000 µL of the sample was injected between the surfaces of two pieces of sausage skin membranes (20 × 40 mm) and then photo‐crosslinked by 405 nm light for 1 min. Two pieces of membranes were then placed into an Instron mechanical tester (Zwick/Roell Z020 with a 1 kN sensor) with a constant peeling speed of 4 mm min^−1^. All measurements were repeated three times.

### Adhesive Strength Test

The adhesive strength was examined using the modified ASTM F2458‐05 standard.^[^ ^46^ ^]^ Fresh porcine skin samples were cut into rectangular sections (20 mm × 40 mm) and pre‐wet with PBS before testing. The two pieces of porcine skin were glued using 1000 µL of the as‐prepared sample and photo‐crosslinked by 405 nm light for 1 min. Two pieces of porcine skin were then placed into an Instron mechanical tester (Zwick/Roell Z020 with a 1 kN sensor) with a strain rate of 4 mm min^−1^. The maximum adhesive strength of each sample was obtained at the point of tearing. All measurements were repeated three times.

### Cytocompatibility

The cytotoxicity test was conducted using the SCFG‐conditioned medium for cell culture.^[^ ^53^ ^]^ To prepare the SCFG‐conditioned medium, the sterilized SCFG (5, 10, 15, 20, 25 mg mL^−1^) was incubated in DMEM supplemented with 10 v/v% fetal bovine serum at 37 °C with a shaking speed of 100 rpm for 24 h. The medium without SCFG was used as a control. L929 cells (ATCC CCL‐1, Cell Bank of the Chinese Academy of Sciences) were seeded in a 96‐well plate at a density of 10 000 cells well^−1^ for 24  h (*n* = 3 per group). Then, the cells were treated with the SCFG‐conditioned medium and incubated at 37 °C for 1 day. The cell viability was evaluated by the Cell Counting Kit‐8 (CCK‐8, Solarbio) method. The media was removed, and CCK‐8 reagent was added and incubated for 1–2 h at 37 °C. The absorbance at 450 nm was measured.

The sterilized SCFG‐incubated medium at a concentration of 10 mg mL^−1^ was chosen to further evaluate the cytocompatibility. L929 cells were seeded in 96‐well plates with a density of 1000 cells/100 µL well^−1^ and pre‐cultured for 24 h at 37 °C in a 5% CO_2_ humidified incubator. Then, the cells were treated with sterilized SCFG‐incubated medium and further incubated for 1, 3, 5, and 7 days (*n* = 3 per group). The cell viability was determined using CCK‐8 assay and Live/Dead assay, respectively. A laser confocal microscope (Leica) was used to image live cells at excitation/emission wavelengths of 490 nm/515 nm, and dead cells at 535 nm/617 nm.

### In Vivo Biocompatibility and Biodegradability

The biocompatibility and biodegradability of SCFG were evaluated by dorsal subcutaneous implantation in a rat model. Male Sprague Dawley rats (SD, 220–250 g) were used for the rat studies. All animals were treated according to the standard guidelines approved by the Institutional Animal Care and Use Committee of the Second Affiliated Hospital, School of Medicine, Zhejiang University (2023#037).

Fibrin glue and SCFG samples were prepared into disk shape using aseptic techniques. The size was 10 mm in diameter and 3 mm in thickness. Rat was anesthetized with 2% sodium pentobarbital. Hair was removed, and the rats were placed over a heating pad during the surgery. The subcutaneous space was accessed by a 1‐cm skin incision per implant in the rat's back. Fibrin glue and SCFG samples were then implanted into the subcutaneous spaces. Up to four implants were placed per rat. The skin incisions were closed with sutures. On days 7, 14, 21, and 28, rats were euthanized and the implanted samples were processed for biodegradation studies. Subcutaneous regions of interest were excised and then fixed in a 4% paraformaldehyde solution for 24 h for histological and immunofluorescence analyses.^[^ ^54^ ^]^

### In Vivo Hemostatic Performance on Rat Models

The hemostatic ability of SCFG was evaluated by both the rat severe liver injury model and the rat heart bleeding model. SD rats (male, 220–250 g) were used for the rat studies. All animals were treated according to the standard guidelines approved by the Institutional Animal Care and Use Committee of the Second Affiliated Hospital, School of Medicine, Zhejiang University (2023#037).

Rat was anesthetized with 2% sodium pentobarbital, and the liver was exposed by an abdominal incision. The pre‐weighed filter paper was placed beneath the liver. A part of the liver lobe (length:3 cm, width:0.5 cm) was cut off, and hemostatic material was applied to the vertical section. The bleeding time was recorded. After 3 min, the filter paper was weighed, and blood loss was calculated. In the control group, no treatment was applied after the liver was cut. Each group contains six rats. In addition, the hemostatic performance of SCFG hydrogel was compared with commonly used tissue adhesives, including Cyanoacrylate (Guangzhou Baiyun Medical Adhesive Company), Surgiflo (Ethicon), Surgicel (Ethicon), Gelatin sponge (Jiangxi Xiangen Medical Technology Co., Ltd.), Combat Gauze (QuikClot), ChitoGauze (HemCon), and Celox (Celox Medical) in a rat severe liver injury model. Each group contains three rats.

Additionally, in vivo, long‐term sealing and biocompatibility of SCFG were evaluated using a rat liver incision bleeding model. Rats were anesthetized with 2% sodium pentobarbital, and the liver was exposed. An incision injury (length:1 cm, depth:0.3 cm) was created using a scalpel. Fibrin glue and SCFG were injected onto the bleeding site. After hemostatic sealing was confirmed, the abdominal incision was closed using sutures. After 2‐week implantation, the rats were euthanized. The livers with the implants were collected and fixed in 4% paraformaldehyde for 24  h for histological and immunofluorescence analyses. The rats were monitored by staff from the Zhejiang University Experimental Animal Center and maintained under normal health conditions.

### In Vivo Hemostasis Ability on Pig Livers

All pigs were treated according to the standard guidelines approved by the Institutional Animal Care and Use Committee of the Second Affiliated Hospital, School of Medicine, Zhejiang University (2023#037). Female Bama pigs (20 kg, Shanghai Jiagan Biotechnology Co., LTD) were used for all in vivo studies in pigs. All animal procedures were performed under general anesthesia. The anesthesia was maintained using propofol (0.1–0.2 mg k^−1^g min^−1^). The liver was exposed, and a pre‐weighed filter paper was placed beneath the liver. Liver incision (length:1.5 cm, depth:0.3 cm) was created. Fibrin glue (*n* = 4) and SCFG (*n* = 4) were injected onto the bleeding site. The time to hemostasis and the blood loss until hemostasis were recorded. After these operations, two pigs were euthanized to collect the liver wound region sealed by the fibrin glue and SCFG, which was used to observe the hemostatic seal between adhesives and the surface of the liver. The remaining pigs were allowed to recover. The abdominal cavity was primarily closed in a layered fashion, and the pigs were woken from general anesthesia. Two weeks after the implantation, the pigs were humanely euthanized, and the wound region sealed by the SCFG was collected and fixed in 4% paraformaldehyde for 24 h for histological analysis. All pigs in the study were monitored by the staff from Zhejiang University Experimental Animal Center to ensure their well‐being.

### Histological Analysis and Immunofluorescence Staining

The tissue samples were fixed with 4% paraformaldehyde for 24 h and then submitted for H&E staining, and immunofluorescence staining. The degree of inflammation was assessed by a certified histopathologist under blinded experimental conditions. The fluorescence intensity of the expressed antibodies was quantified using ImageJ (v.1.5.2).

### Statistical Analysis

All data were presented as mean ± standard deviation (SD). For comparison between multiple groups, a one‐way ANOVA followed by post‐hoc analysis (Tukey's test) was used. For comparison between the two groups, a two‐sided Student's t‐test was performed. P‐values less than 0.05 were considered statistically significant.

## Conflict of Interest

Zhejiang University has filed for patent protection on the materials and method described herein, and Z.M., L.Y., W.W., and Y.D. are named as inventors on the patent (application number:202 211 249 717.0). The remaining authors declare no conflict of interest.

## Author Contributions

L.Y. and Z.L. contributed equally to this work. Z.M., Y.D., W.W., L.Y., and Z.L. conceived the idea and designed the study. L.Y., Z.L., and Z.T. carried out the in vitro experiments. L.Y., Z.L., and Z.Q. performed the in vivo studies. Z.M., Y.D., L.Y., and Z.L. analyzed and interpreted the results. Z.M., Y.D., W.W., L.Y., and Z.L. wrote the manuscript with inputs from all authors.

## Supporting information

Supporting Information

Supplemental Movie 1

Supplemental Movie 2

Supplemental Movie 3

## Data Availability

The data that support the findings of this study are available from the corresponding author upon reasonable request.

## References

[1] J. W. Cannon , N. Engl. J. Med. 2018, 378, 370. 29365303 10.1056/NEJMra1705649

[2] X. Zhao , B. Guo , H. Wu , Y. Liang , P. X. Ma , Nat. Commun. 2018, 9, 2784. 30018305 10.1038/s41467-018-04998-9 PMC6050275

[3] R. Lozano , M. Naghavi , K. Foreman , S. Lim , K. Shibuya , V. Aboyans , J. Abraham , T. Adair , R. Aggarwal , S. Y. Ahn , M. A. Almazroa , M. Alvarado , H. R. Anderson , L. M. Anderson , K. G. Andrews , C. Atkinson , L. M. Baddour , S. Barker‐Collo , D. H. Bartels , M. L. Bell , E. J. Benjamin , D. Bennett , K. Bhalla , B. Bikbov , A. B. Abdulhak , G. Birbeck , F. Blyth , I. Bolliger , S. Boufous , C. Bucello , et al., Lancet 2012, 380, 2095.23245604

[4] D. A. Hickman , C. L. Pawlowski , U. D. S. Sekhon , J. Marks , A. S. Gupta , Adv. Mater. 2018, 30, 1700859. 10.1002/adma.201700859 PMC5831165 29164804

[5] H. Wang , J. Cheng , F. Sun , X. Dou , J. Liu , Y. Wang , M. Li , J. Gao , X. Liu , X. Wang , F. Yang , Z. Zhu , H. Shen , L. Zhang , P. Tang , D. Wu , Adv. Mater. 2022, 35, 2208622. 10.1002/adma.202208622 36579739

[6] D. R. King , N. Engl. J. Med. 2019, 380, 763. 30786189 10.1056/NEJMra1609326

[7] B. Guo , R. Dong , Y. Liang , M. Li , Nat. Rev. Chem. 2021, 5, 773. 37117664 10.1038/s41570-021-00323-z

[8] S. Pourshahrestani , E. Zeimaran , I. Djordjevic , N. A. Kadri , M. R. Towler , Mater. Sci. Eng. C. 2016, 58, 1255. 10.1016/j.msec.2015.09.008 26478429

[9] X. Shang , H. Chen , V. Castagnola , K. Liu , L. Boselli , V. Petseva , L. Yu , L. Xiao , M. He , F. Wang , K. A. Dawson , J. Fan , Nat. Catal. 2021, 4, 607.

[10] R. Dong , H. Zhang , B. Guo , Natl Sci Rev 2022, 9, nwac162. 36381219 10.1093/nsr/nwac162 PMC9646998

[11] X. Du , L. Wu , H. Yan , Z. Jiang , S. Li , W. Li , Y. Bai , H. Wang , Z. Cheng , D. Kong , L. Wang , M. Zhu , Nat. Commun. 2021, 12, 4733. 34354068 10.1038/s41467-021-24972-2 PMC8342549

[12] H. Yuk , J. Wu , T. L. Sarrafian , X. Mao , C. E. Varela , E. T. Roche , L. G. Griffiths , C. S. Nabzdyk , X. Zhao , Nat. Biomed. Eng. 2021, 5, 1131. 34373600 10.1038/s41551-021-00769-y PMC9254891

[13] G. Bao , Q. Gao , M. Cau , N. Ali‐Mohamad , M. Strong , S. Jiang , Z. Yang , A. Valiei , Z. Ma , M. Amabili , Z.‐H. Gao , L. Mongeau , C. Kastrup , J. Li , Nat. Commun. 2022, 13, 5035. 36028516 10.1038/s41467-022-32803-1 PMC9418157

[14] S. O. Blacklow , J. Li , B. R. Freedman , M. Zeidi , C. Chen , D. J. Mooney , Sci. Adv. 2019, 5, eaaw3963. 31355332 10.1126/sciadv.aaw3963 PMC6656537

[15] C. Cui , C. Fan , Y. Wu , M. Xiao , T. Wu , D. Zhang , X. Chen , B. Liu , Z. Xu , B. Qu , W. Liu , Adv. Mater. 2019, 31, 1905761. 10.1002/adma.201905761 31625635

[16] J. Li , A. D. Celiz , J. Yang , Q. Yang , I. Wamala , W. Whyte , B. R. Seo , N. V. Vasilyev , J. J. Vlassak , Z. Suo , D. J. Mooney , Science 2017, 357, 378. 28751604 10.1126/science.aah6362 PMC5905340

[17] C. Wei , W. Shi , C. Zhao , S. Yang , J. Zheng , J. Zhong , T. Zhao , S. Kong , X. Gong , M. Liu , Adv. Healthcare Mater. 2022, 12, e2201799. 10.1002/adhm.202201799 36333905

[18] Z. Qiao , X. Lv , S. He , S. Bai , X. Liu , L. Hou , J. He , D. Tong , R. Ruan , J. Zhang , J. Ding , H. Yang , Bioact Mater 2021, 6, 2829. 33718665 10.1016/j.bioactmat.2021.01.039 PMC7905459

[19] H. Yuk , C. E. Varela , C. S. Nabzdyk , X. Mao , R. F. Padera , E. T. Roche , X. Zhao , Nature 2019, 575, 169. 31666696 10.1038/s41586-019-1710-5

[20] Z. Ma , G. Bao , J. Li , Adv. Mater. 2021, 33, 2007663. 10.1002/adma.202007663 33956371

[21] S. Nam , D. Mooney , Chem. Rev. 2021, 121, 11336. 33507740 10.1021/acs.chemrev.0c00798

[22] G. M. Taboada , K. Yang , M. J. N. Pereira , S. S. Liu , Y. Hu , J. M. Karp , N. Artzi , Y. Lee , Nat. Rev. Mater. 2020, 5, 310.

[23] Y. Hong , F. Zhou , Y. Hua , X. Zhang , C. Ni , D. Pan , Y. Zhang , D. Jiang , L. Yang , Q. Lin , Y. Zou , D. Yu , D. E. Arnot , X. Zou , L. Zhu , S. Zhang , H. Ouyang , Nat. Commun. 2019, 10, 2060. 31089131 10.1038/s41467-019-10004-7 PMC6517429

[24] B. Xue , J. Gu , L. Li , W. Yu , S. Yin , M. Qin , Q. Jiang , W. Wang , Y. Cao , Nat. Commun. 2021, 12, 7156. 34887418 10.1038/s41467-021-27529-5 PMC8660897

[25] B. Kong , R. Liu , Y. Cheng , Y. Shang , D. Zhang , H. Gu , Y. Zhao , W. Xu , Adv. Sci. 2022, 9, e2203096. 10.1002/advs.202203096 PMC9631070 36089655

[26] E. E. Leonhardt , N. Kang , M. A. Hamad , K. L. Wooley , M. Elsabahy , Nat. Commun. 2019, 10, 2307. 31127114 10.1038/s41467-019-10290-1 PMC6534699

[27] G. Wang , X. Meng , P. Wang , X. Wang , G. Liu , D.‐A. Wang , C. Fan , Biomaterials 2022, 291, 121908. 36384085 10.1016/j.biomaterials.2022.121908

[28] Y. Gao , K. Peng , S. Mitragotri , Adv. Mater. 2021, 33, 2006362. 10.1002/adma.202006362 33988273

[29] X. Zhao , B. Guo , H. Wu , Y. Liang , P. X. Ma , Nat. Commun. 2018, 9, 2784. 30018305 10.1038/s41467-018-04998-9 PMC6050275

[30] W. D. Spotnitz , World J. Surg. 2010, 34, 632. 19820991 10.1007/s00268-009-0252-7

[31] S. P. Mandell , N. S. Gibran , Expert Opin. Biol. Ther. 2014, 14, 821. 24625330 10.1517/14712598.2014.897323

[32] M. Beudert , M. Gutmann , T. Lühmann , L. Meinel , ACS Biomater. Sci. Eng. 2022, 8, 2220. 35610572 10.1021/acsbiomaterials.1c01437

[33] H. Khoshmohabat , S. Paydar , H. M. Kazemi , B. Dalfardi , Rev. Ortop. Traumatol. Latinoam. 2016, 21, e26023. 10.5812/traumamon.26023 PMC4869418 27218055

[34] M. N. Sundaram , V. Krishnamoorthi Kaliannagounder , V. Selvaprithiviraj , M. K. Suresh , R. Biswas , A. K. Vasudevan , P. K. Varma , R. Jayakumar , ACS Sustainable Chem. Eng. 2018, 6, 7826.

[35] Y. Yang , X. Wang , F. Yang , H. Shen , D. Wu , Adv. Mater. 2016, 28, 7178. 27301068 10.1002/adma.201601742

[36] T. Matsuda , T. Nakajima , J. P. Gong , Chem. Mater. 2019, 31, 3766.

[37] R. Haghniaz , H. Montazerian , A. Rabbani , A. Baidya , B. Usui , Y. Zhu , M. Tavafoghi , F. Wahid , H.‐J. Kim , A. Sheikhi , A. Khademhosseini , Adv. Healthcare Mater. 2023, 10.1002/adhm.202301551. PMC10710521 37300448

[38] Y. Guo , Y. Wang , X. Zhao , X. Li , Q. Wang , W. Zhong , K. Mequanint , R. Zhan , M. Xing , G. Luo , Sci. Adv. 2021, 7, eabf9635. 34261653 10.1126/sciadv.abf9635 PMC8279511

[39] J. W. Nichol , S. T. Koshy , H. Bae , C. M. Hwang , S. Yamanlar , A. Khademhosseini , Biomaterials 2010, 31, 5536. 20417964 10.1016/j.biomaterials.2010.03.064 PMC2878615

[40] X. Zhao , Q. Lang , L. Yildirimer , Z. Y. Lin , W. Cui , N. Annabi , K. W. Ng , M. R. Dokmeci , A. M. Ghaemmaghami , A. Khademhosseini , Adv. Healthcare Mater. 2016, 5, 108. 10.1002/adhm.201500005 PMC4608855 25880725

[41] A. Assmann , A. Vegh , M. Ghasemi‐Rad , S. Bagherifard , G. Cheng , E. S. Sani , G. U. Ruiz‐Esparza , I. Noshadi , A. D. Lassaletta , S. Gangadharan , A. Tamayol , A. Khademhosseini , N. Annabi , Biomaterials 2017, 140, 115. 28646685 10.1016/j.biomaterials.2017.06.004 PMC5993547

[42] D. Loessner , C. Meinert , E. Kaemmerer , L. C. Martine , K. Yue , P. A. Levett , T. J. Klein , F. P. W. Melchels , A. Khademhosseini , D. W. Hutmacher , Nat. Protoc. 2016, 11, 727. 26985572 10.1038/nprot.2016.037

[43] I. Noshadi , S. Hong , K. E. Sullivan , E. Shirzaei Sani , R. Portillo‐Lara , A. Tamayol , S. R. Shin , A. E. Gao , W. L. Stoppel , L. D. Black Iii , A. Khademhosseini , N. Annabi , Biomater. Sci. 2017, 5, 2093. 28805830 10.1039/c7bm00110j PMC5614854

[44] X. Xu , X. Xia , K. Zhang , A. Rai , Z. Li , P. Zhao , K. Wei , L. Zou , B. Yang , W.‐K. Wong , P. W.‐Y. Chiu , L. Bian , Sci. Transl. Med. 2020, 12, eaba8014. 32848095 10.1126/scitranslmed.aba8014

[45] S. Taheri , G. Bao , Z. He , S. Mohammadi , H. Ravanbakhsh , L. Lessard , J. Li , L. Mongeau , Adv. Sci. 2022, 9, e2102627. 10.1002/advs.202102627 PMC8805581 34811970

[46] N. Annabi , Y.‐N. Zhang , A. Assmann , E. S. Sani , G. Cheng , A. D. Lassaletta , A. Vegh , B. Dehghani , G. U. Ruiz‐Esparza , X. Wang , S. Gangadharan , A. S. Weiss , A. Khademhosseini , Sci. Transl. Med. 2017, 9, eaai7466. 28978753 10.1126/scitranslmed.aai7466 PMC11186511

[47] T. Deng , D. Gao , X. Song , Z. Zhou , L. Zhou , M. Tao , Z. Jiang , L. Yang , L. Luo , A. Zhou , L. Hu , H. Qin , M. Wu , Nat. Commun. 2023, 14, 396. 36693849 10.1038/s41467-023-35907-4 PMC9873654

[48] X. Peng , X. Xia , X. Xu , X. Yang , B. Yang , P. Zhao , W. Yuan , P. W. Y. Chiu , L. Bian , Sci. Adv. 2021, 7, eabe8739. 34078598 10.1126/sciadv.abe8739 PMC8172133

[49] X. Peng , X. Xu , Y. Deng , X. Xie , L. Xu , X. Xu , W. Yuan , B. Yang , X. Yang , X. Xia , L. Duan , L. Bian , Adv. Funct. Mater. 2021, 31, 2102583.

[50] L. Montanaro , C. R. Arciola , E. Cenni , G. Ciapetti , F. Savioli , F. Filippini , L. A. Barsanti , Biomaterials 2001, 22, 59. 11085384 10.1016/s0142-9612(00)00163-0

[51] A. J. Singer , J. V. Quinn , J. E. Hollander , Am J Emerg Med 2008, 26, 490. 18410821 10.1016/j.ajem.2007.05.015

[52] J. Zhang , B. Gao , B. Ye , Z. Sun , Z. Qian , L. Yu , Y. Bi , L. Ma , Y. Ding , Y. Du , W. Wang , Z. Mao , Adv. Mater. 2023, 35, 2208571. 10.1002/adma.202208571 36648306

[53] L. Jin , F. Cao , Y. Gao , C. Zhang , Z. Qian , J. Zhang , Z. Mao , Adv. Mater. 2023, 35, 2301349. 10.1002/adma.202301349 37083074

[54] F. Cao , L. Jin , Y. Gao , Y. Ding , H. Wen , Z. Qian , C. Zhang , L. Hong , H. Yang , J. Zhang , Z. Tong , W. Wang , X. Chen , Z. Mao , Nat. Nanotechnol. 2023, 18, 617. 36973397 10.1038/s41565-023-01346-x

