# Supplementary material for: Sequential‐Crosslinking Fibrin Glue for Rapid and Reinforced Hemostasis
Source: Adv Sci (Weinh). 2023 Dec 10;11(7):2308171. doi: 10.1002/advs.202308171 (PMC10870078; doi:10.1002/advs.202308171)
Supplement: Supplementary file 1 — Supporting Information [file ADVS-11-2308171-s001.pdf]

ADVANCED SCIENCE
Open Access

Supporting Information

for *Adv. Sci.*, DOI 10.1002/advs.202308171

Sequential-Crosslinking Fibrin Glue for Rapid and Reinforced Hemostasis

Lisha Yu, Zhaodi Liu, Zongrui Tong, Yihang Ding, Zhefeng Qian, Weilin Wang, Zhengwei Mao* and Yuan Ding**

Supplementary Materials

Sequential-crosslinking fibrin glue for rapid and reinforced hemostasis

Lisha Yu[1,3#], Zhaodi Liu[1,3,4,5,6,7#], Zongrui Tong[1,3], Yihang Ding[2], Zhefeng Qian[1,2,3], Weilin Wang[1,3,4,5,6,7*], Zhengwei Mao[1,2,3*], Yuan Ding[1,3,4,5,6,7*]

[1] Department of Hepatobiliary and Pancreatic Surgery, The Second Affiliated Hospital, School of Medicine, Zhejiang University, Zhejiang, Hangzhou, 310009, China

[2] MOE Key Laboratory of Macromolecular Synthesis and Functionalization, Department of Polymer Science and Engineering, Zhejiang University, Zhejiang, Hangzhou, 310027, China

[3] Key Laboratory of Precision Diagnosis and Treatment for Hepatobiliary and Pancreatic Tumor of Zhejiang Province, Zhejiang, Hangzhou, 310009, China

[4] Research Center of Diagnosis and Treatment Technology for Hepatocellular Carcinoma of Zhejiang Province, Zhejiang, Hangzhou, 310009, China

[5] National Innovation Center for Fundamental Research on Cancer Medicine, Zhejiang, Hangzhou, 310009, China

[6] Cancer Center, Zhejiang University, Zhejiang, Hangzhou, 310058, China

[7] ZJU-Pujian Research & Development Center of Medical Artificial Intelligence for Hepatobiliary and Pancreatic Disease, Zhejiang, Hangzhou, 310058, China

[#] Lisha Yu and Zhaodi Liu contributed equally to this work.

*Correspondence and requests for materials should be addressed to Weilin Wang (wam@zju.edu.cn), Zhengwei Mao (zwmao@zju.edu.cn) and Yuan Ding (dingyuan@zju.edu.cn).

Supplementary Movie 1. The sequential crosslinking formation of SCFG.

Supplementary Movie 2. In vivo hemostasis test of fibrin glue in a rat severe liver injury model.

Supplementary Movie 3. In vivo hemostasis test of SCFG in a rat severe liver injury model.

Supplementary materials and methods

Materials

Alginate methacryloyl (AlgMA, 50kDa), hyaluronic acid methacryloyl (HAMA, 150kDa), silk fibroin methacryloyl (SilMA), chitosan methacryloyl (CSMA, 100kDa) and poly (ethylene glycol) diacrylate (PEGDA, 1kD) were obtained from Suzhou Yongqinquan Intelligent Equipment Co., Ltd. (Suzhou, China).

Rheological study

Rheological property was performed using a rotary rheometer (HAAKE RS6000) with parallel plate (P20 TiL, 20-mm diameter) geometry. The as-prepared samples (20 mm in diameter and 2 mm in thickness) were loaded on the parallel plate. Time-sweep oscillatory test was performed at a 5% strain and 1 Hz frequency at 37 °C. The storage modulus (G′) and loss modulus (G″) were recorded.

Gelation time assay

The gelation time is the period required from liquid solution to the appearance of hydrogel. It can be determined with a vial tilting method[1]. The hydrogel precursors are mixed in a vial, and the time when no flow is observed after tilting the vial is recorded as the gelation time. Because the sealants in clinical settings will come into contact with blood, we also evaluate the gelation time in the presence of blood. The hydrogel precursors are mixed in a blood-covered tube, and the gelation time was recorded.

Storage stability

Precursor solutions of GelMA/Fibrinogen (GelMA/Fg) and GelMA/Thrombin (GelMA/Thr) were prepared and stored at room temperature. Gelation time and rheological analysis of SCFG from the precursor solution for storage duration was performed to evaluate the storage stability of precursor solution.

Tensile test

To measure the tensile behavior[2,3], the hydrogel was cut into strips of length 35 mm, width 15 mm, and thickness 3 mm, and tested with an Instron mechanical tester (Zwick/Roell Z020 with a 1 kN sensor) with a strain rate of 50 mm/min. The initial height (the distance between the two clamps) of the hydrogel specimen was 15 mm. The nominal (engineering) stress was obtained by dividing the force by the initial cross-sectional area. The nominal (engineering) strain was

obtained by dividing the deformed length by the initial length. Tensile strength was determined by dividing the maximum force by the cross-sectional area. All measurements were repeated three times.

Fracture toughness measurement

The fracture toughness of hydrogels was measured using pure shear tests. A razor blade was used to create a notch of 3 mm starting from the edge of the identical specimens (15 mm in width, 35 mm in height, and 3 mm in thickness). The height (H) of the specimen (the distance between the two clamps) was 15 mm. The notched sample was pulled until rupture to obtain a critical stretch (λ_c). The fracture energy was calculated from S-λ curve of the unnotched sample by[2]

$$\Gamma = H \int_1^{\lambda_c} S d\lambda \qquad \text{Equation 1}$$

Swelling ratio

Samples were incubated in PBS at 37 °C for 24 h, then blotted dry with filter paper to remove free liquid and recorded the weight as W_s. After lyophilization, the dry weight of hydrogels was recorded as W_d. The swelling ratio (SR) was calculated according to Equation 2. Three samples were repeated for each group.

$$SR = (W_s - W_d)/W_d \qquad \text{Equation 2}$$

Burst pressure test

Burst pressure testing was performed using a published method[3]. Briefly, a piece of 4 × 4 cm porcine skin was prepared and tested using the standard burst pressure test (ASTM F2392-04). A 3-mm hole was introduced to the porcine skin. Then, 500 μL of precursor solutions were injected onto porcine skin and covered the hole, after which the hydrogels formed in situ on the puncture site after UV illumination. The thickness of the hydrogels was 4 mm and burst pressure was measured after gel formation. The pressure was applied to the sealed porcine skin by pumping air using a syringe pump at a flow rate of 2 mL min^{-1}. The maximum pressure was recorded as the burst pressure using a digital pressure gauge. All experiments were repeated three times.

Preparation of SCFG by various widely used biopolymer gels

A series of sequential crosslinked fibrin glue were also prepared using AlgMA, HAMA, SilMA,

CSMA and PEGDA. For SCFG composed of AlgMA, the AlgMA/thrombin solution was with the concentration of 7.5%(w/v) AlgMA, 1000 IU/mL thrombin, and 0.25%(w/v) LAP. The AlgMA/fibrinogen solution was the concentration of 2.5%(w/v) AlgMA, 5%(w/v) fibrinogen, and 0.125%(w/v) LAP. The final concentration of AlgMA in SCFG was 5%(w/v)AlgMA. In other sequential crosslinked fibrin glue, the final concentration of HAMA, SilMA, CSMA and PEGDA were 3.75%(w/v), 8%(w/v), 1.25%(w/v), 10%(w/v), respectively.

***In vivo* hemostatic performance on rat hearts**

All animals were treated according to the standard guidelines approved by the Institutional Animal Care and Use Committee of the Second Affiliated Hospital, School of Medicine, Zhejiang University (2023#037). In the rat heart bleeding model, rat was anesthetized with 2% sodium pentobarbital. And heart was exposed via a thoracotomy. A 2.4 mm diameter injury was made on rat heart, leading to acute bleeding. Fibrin glue and SCFG were immediately injected onto the bleeding site. After the hemostatic sealing was confirmed, the time to hemostasis was recorded.

***In vivo* hemostasis performance on rabbit livers**

All animals were treated according to the standard guidelines approved by the Institutional Animal Care and Use Committee of the Second Affiliated Hospital, School of Medicine, Zhejiang University (2023#037). Female New Zealand white rabbits (2.5-3.0 kg) were used for in vivo studies in rabbits. The hemostatic performance of SCFG were evaluated using both rabbit liver penetration model and hepatectomy model. The liver incision (length: 1 cm, depth: 0.5 cm) was carried out in rabbit liver penetration model. The data of bleeding time and blood loss were recorded after 3 min of the hemostatic process. Each group consisted of ten rabbits. A part of the liver lobe (length: 3 cm, width: 0.5 cm) was cut off in rabbit hepatectomy model. The bleeding time and blood loss were also recorded after 3 min of the hemostatic process. Each group contained eight rabbits.

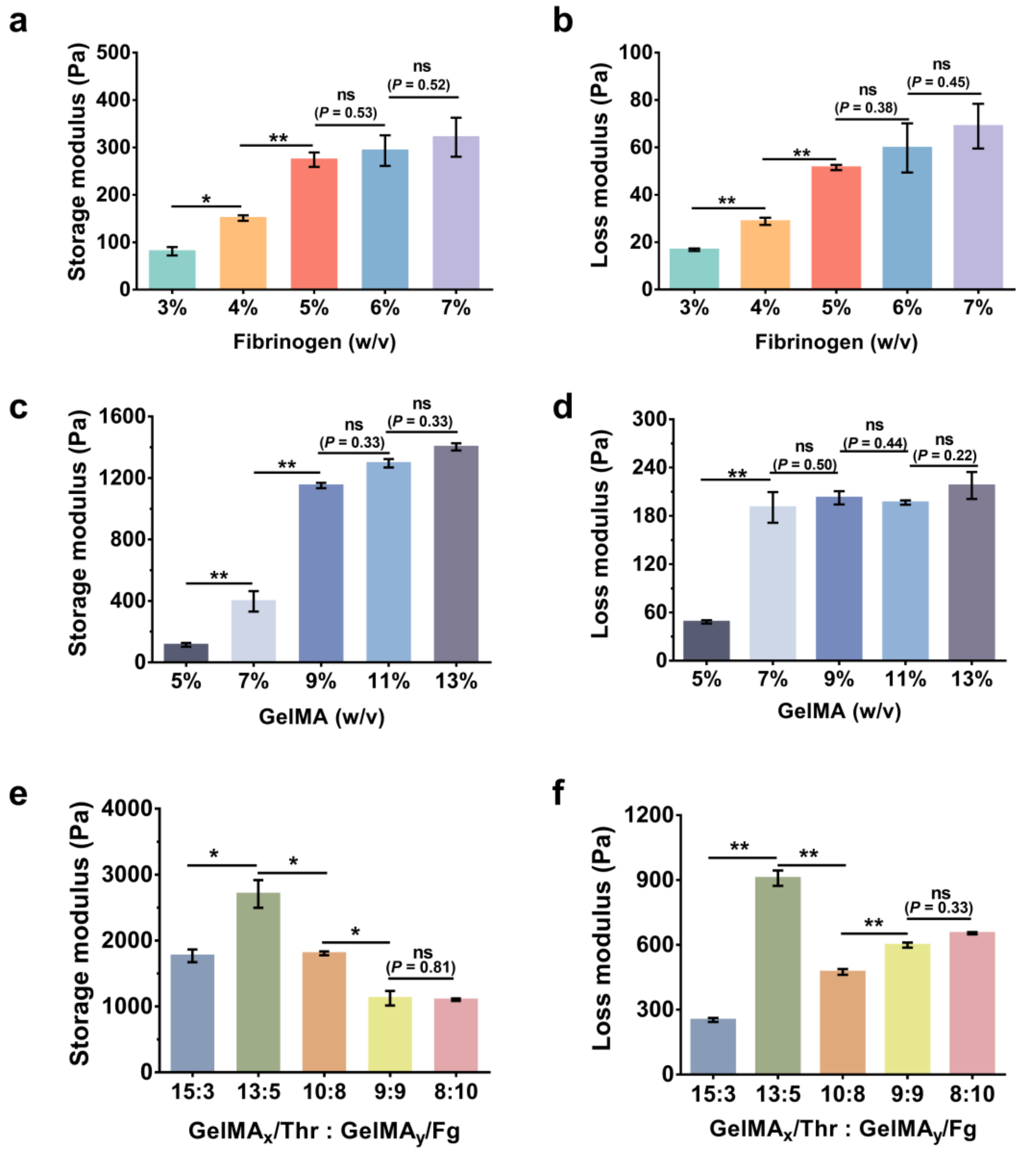

Figure S1 Composition of SCFG on the mechanical properties. (**a**) Storage modulus G' and (**b**) loss modulus G" of fibrin glue with different concentration of fibrinogen. (**c**) Storage modulus G' and (**d**) loss modulus G" of GelMA with different concentration. (**e**) Storage modulus G' and (**f**) loss modulus G" of SCFG with composition ratios (x:y) of GelMA for preparing $GelMA_x$/Thr and $GelMA_y$/Fg. The maximum storage modulus G' and loss modulus G" of SCFG was at the composition ratios of 13% (w/v) GelMA/Thr and 5% (w/v) GelMA/Fg. P values are determined by two-sided t test. Error bars, mean ± SD. ns. not significant, $*p < 0.05$; $**p < 0.01$.

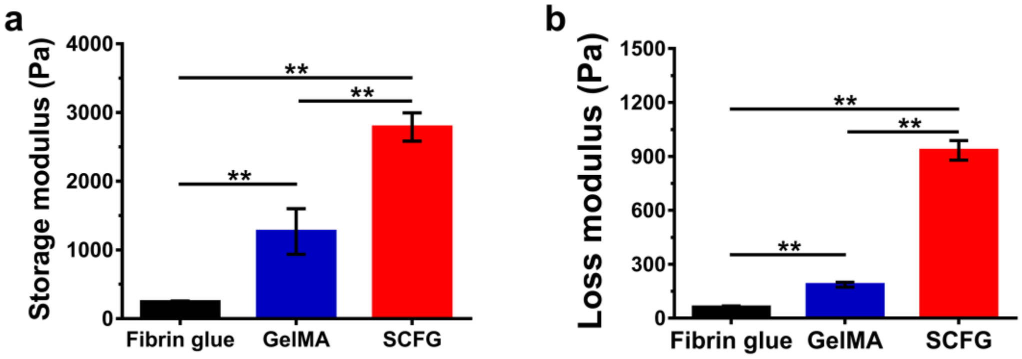

Figure S2 (**a**) Storage modulus and (**b**) loss modulus of fibrin glue, GelMA and SCFG. P values are determined by one-way ANOVA followed by the Tukey's comparison test. Error bars, mean ± SD. $^{**}p < 0.01$.

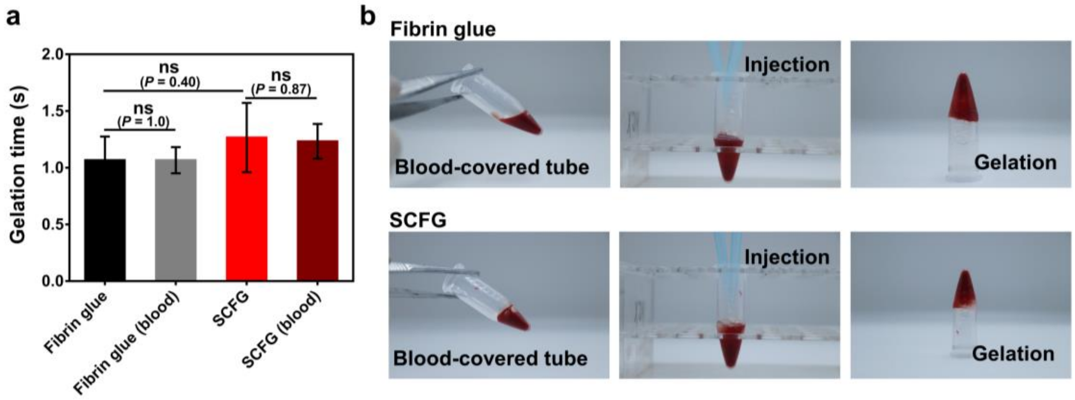

Figure S3 a, Gelation time of fibrin glue and SCFG. **b,** Photos of hydrogel formation in blood-covered tube, and the presence of blood does not affect the gelation time. P values are determined by two-sided t test. Error bars, mean ± SD. ns. not significant.

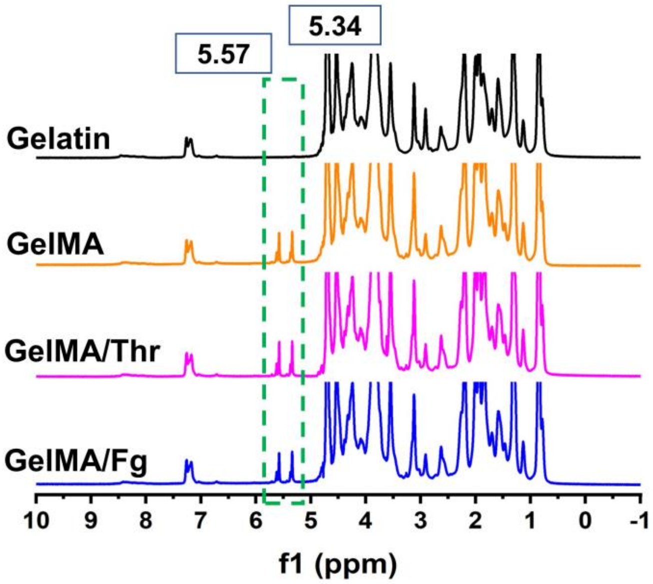

Figure S4 ^1H NMR spectra of gelatin, GelMA, GelMA/Thr, and GelMA/Fg in D_2O at room temperature. The ^1H nuclear magnetic resonance (^1H NMR) clearly demonstrated the original methacrylic groups of GelMA in the SCFG precursor solution, ensuring light-induced GelMA polymerization.

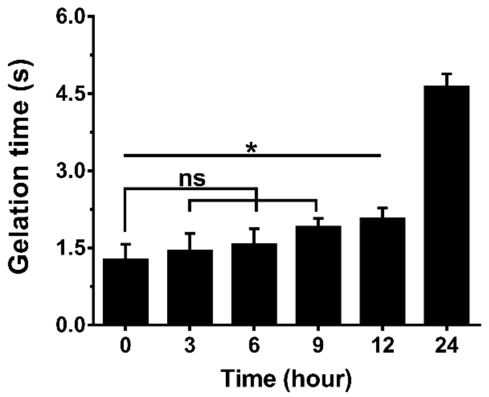

Figure S5 Gelation time of SCFG from the precursor solution for storage duration at room temperature. P values are determined by one-way ANOVA followed by the Tukey's comparison test. Error bars, mean ± SD. ns. not significant, $*p < 0.05$.

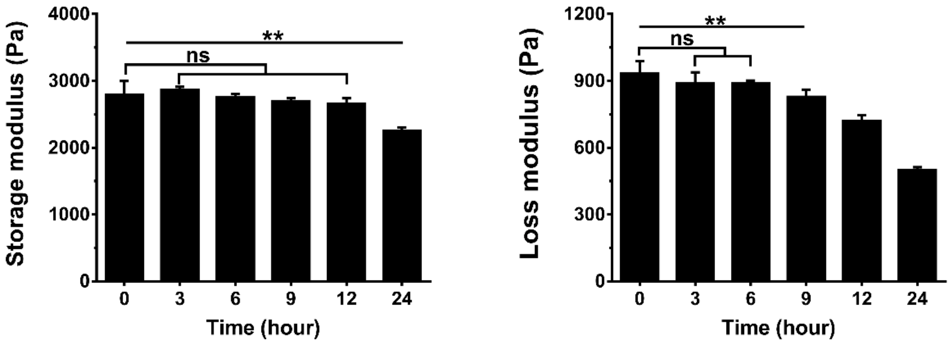

Figure S6 Storage effect of the precursor solution at room temperature. (**a**) Storage modulus (G') and (**b**) loss modulus (G") of SCFG generated from the precursor solution for storage duration. P values are determined by one-way ANOVA followed by the Tukey's comparison test. Error bars, mean ± SD. ns. not significant, **$p < 0.01$.

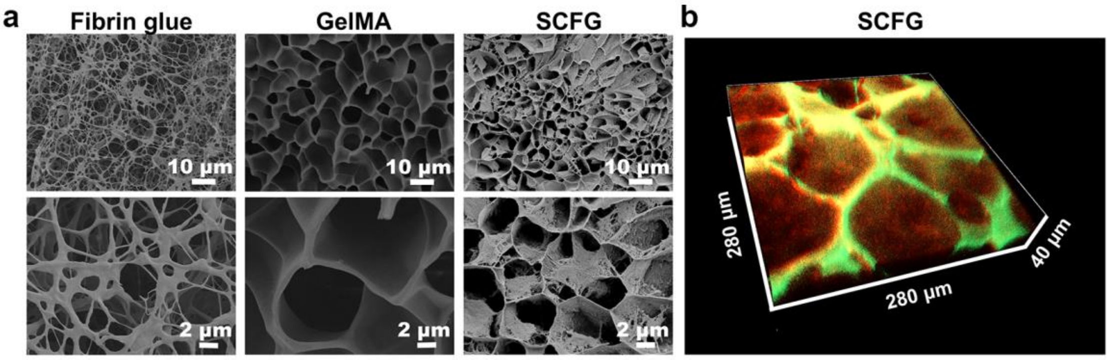

Figure S7 a, SEM images of fibrin glue, GelMA, and SCFG after the freeze-drying treatment, respectively. **b,** CLSM three-dimensional image of SCFG. This image is constructed from z-stacked x–y slice images, in which FITC-labeled GelMA (green) and Cyanine 3-labeled fibrin mesh (red) were interpenetrated.

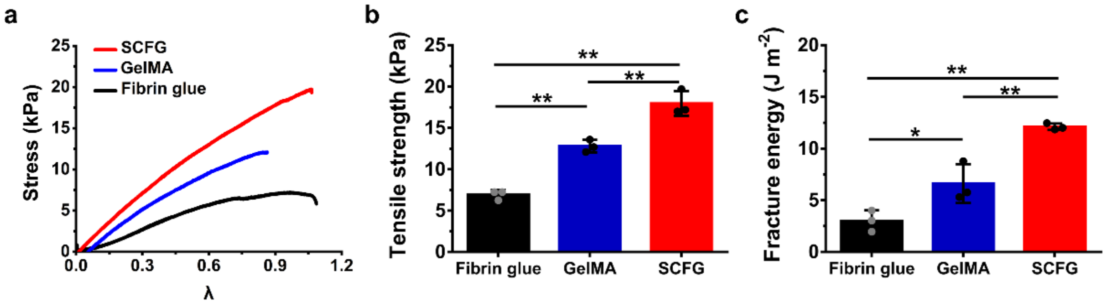

Figure S8 Tensile strength of SCFG. **a,** Stress-stretch curves of the tensile test. **b,** Tensile strength of fibrin glue, GelMA and SCFG, respectively. **c,** Fracture energy of fibrin glue, GelMA and SCFG. P values are determined by one-way ANOVA followed by the Tukey's comparison test. Error bars, mean ± SD. $*p < 0.05$; $**p < 0.01$.

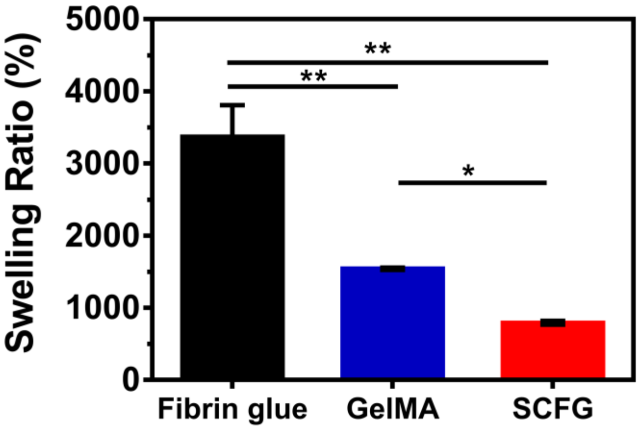

Figure S9 Swelling ratio of fibrin glue, GelMA and SCFG after 24 h incubation in PBS at 37 °C. P values are determined by one-way ANOVA followed by the Tukey's comparison test. Error bars, mean ± SD. *$p < 0.05$; **$p < 0.01$.

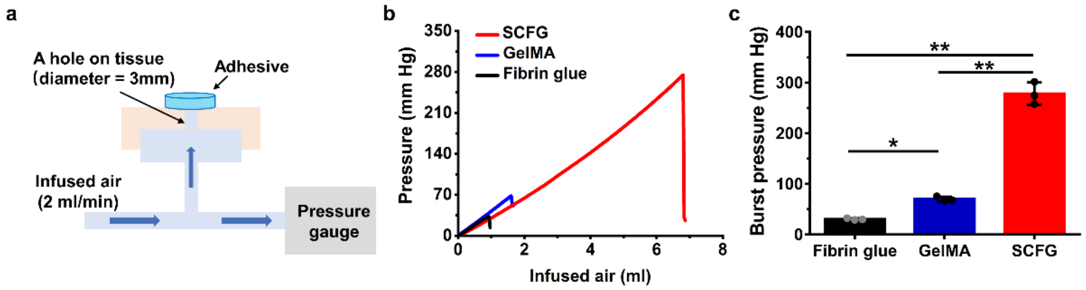

Figure S10 Burst pressure of SCFG. a, Schematic diagram of measuring burst pressure. **b,** Pressure–Infused air curves of the burst pressure test. **c,** Burst pressure of fibrin glue, GelMA and SCFG. P values are determined by one-way ANOVA followed by the Tukey's comparison test. Error bars, mean ± SD. $*p < 0.05$; $**p < 0.01$.

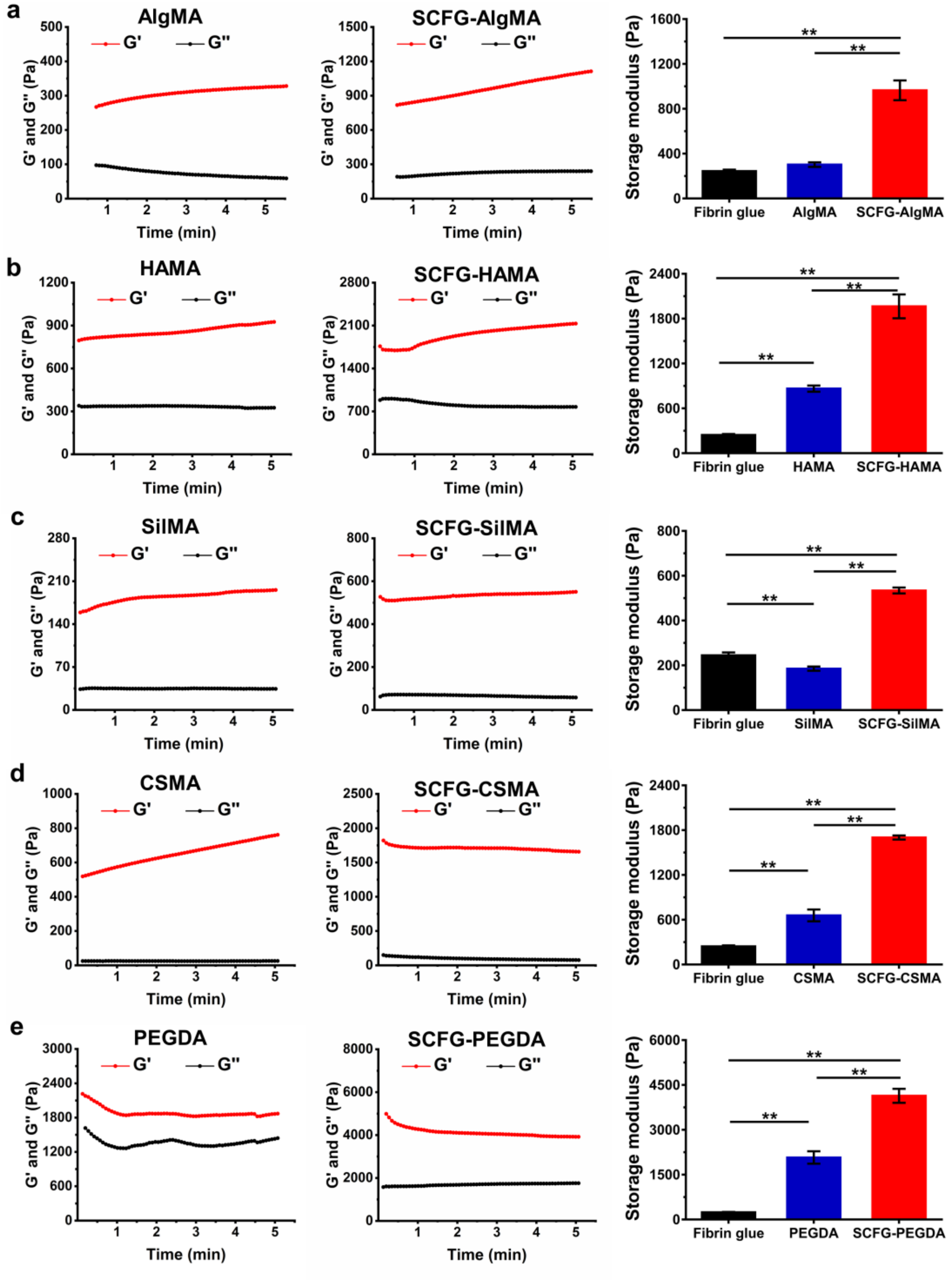

Figure S11 Storage modulus and loss modulus of SCFG composed by (a) AlgMA, (b) HAMA, (c) SilMA, (d) CSMA and (e) PEGDA, respectively. P values are determined by one-way ANOVA followed by the Tukey's comparison test. Error bars, mean ± SD. $*p < 0.05$; $**p < 0.01$.

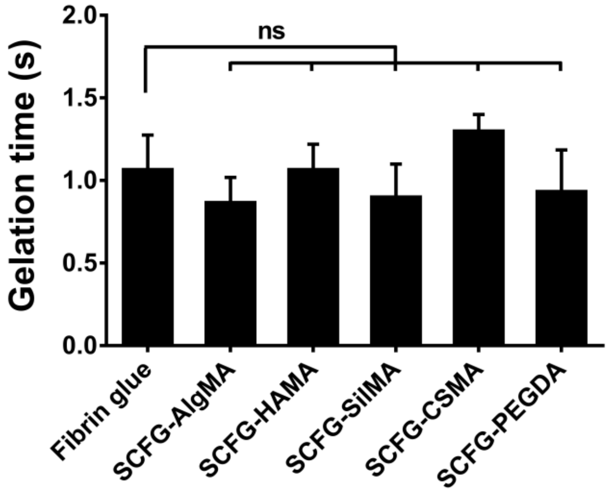

Figure S12 Gelation time of SCFG composed by AlgMA, HAMA, SilMA, CSMA and PEGDA, respectively. P values are determined by two-sided t test. Error bars, mean ± SD. ns. not significant.

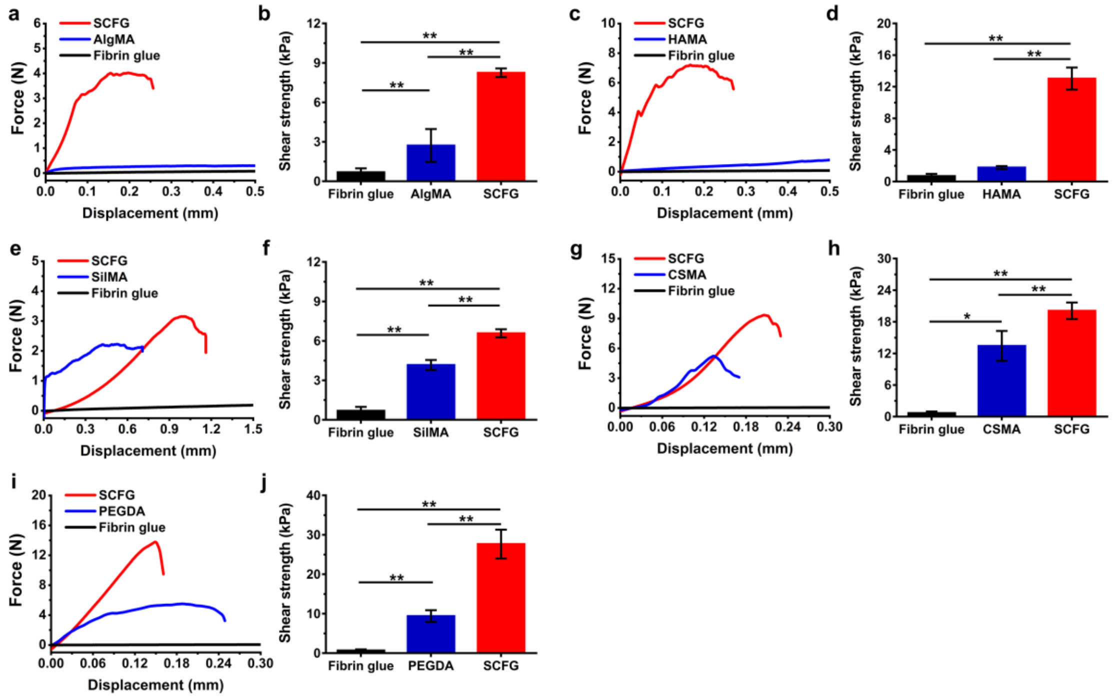

Figure S13 Force-displacement curves of the Lap-shear test and shear strength of SCFG composed by (a,b) AlgMA, (c,d) HAMA, (e,f) SilMA, (g,h) CSMA and (i,j) PEGDA, respectively. P values are determined by one-way ANOVA followed by the Tukey's comparison test. Error bars, mean ± SD. $*p < 0.05$; $**p < 0.01$.

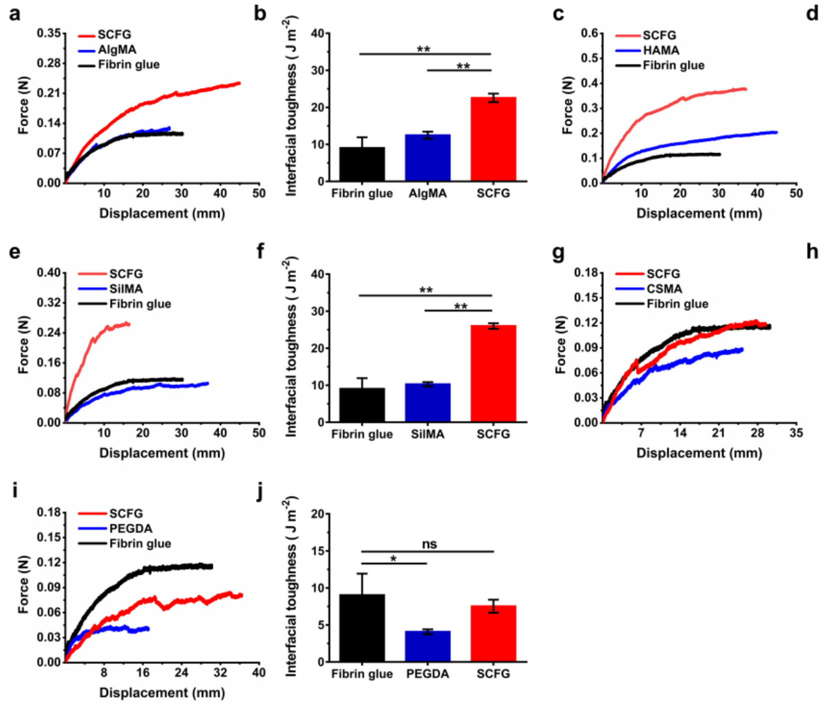

Figure S14 Force-displacement curves of T-peel adhesion test and interfacial toughness of SCFG composed by (a,b) AlgMA, (c,d) HAMA, (e,f) SilMA, (g,h) CSMA and (i,j) PEGDA, respectively. P values are determined by one-way ANOVA followed by the Tukey's comparison test. Error bars, mean ± SD. ns. not significant, $*p < 0.05$; $**p < 0.01$.

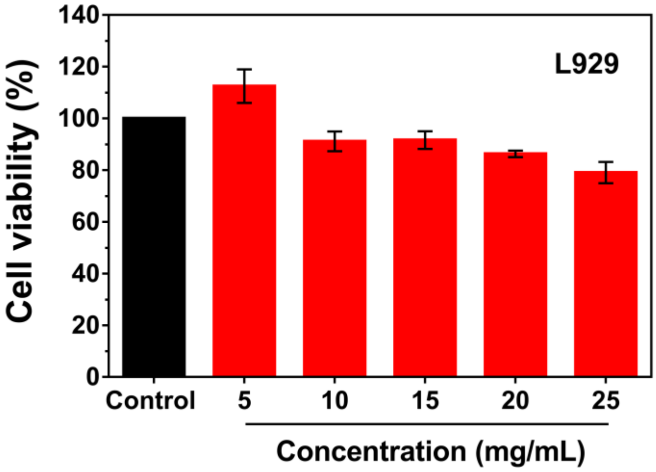

Figure S15 Cytotoxicity of SCFG. In vitro L929 cell in control media and SCFG-incubated media with different concentrations (5, 10, 15, 20 and 25 mg/mL) after 24 h incubation. Error bars, Mean ± SD, n = 3.

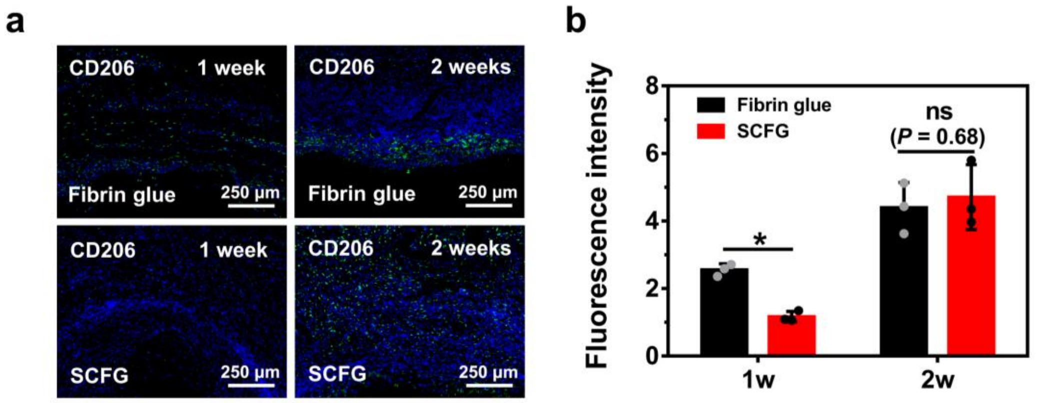

Figure S16 Immunofluorescence analysis of the dorsal subcutaneous implantation. a, Representative immunofluorescence images of M2 type macrophage (CD206) makers for fibrin glue and SCFG at 1 week and 2 weeks. **b,** Fluorescence intensity from the immunofluorescence images of M2 type macrophage (CD206) makers for fibrin glue and SCFG at 1 week and 2 weeks (n = 3). P values are determined by two-sided t test. Error bars, mean ± SD. *p < 0.05; ns. not significant.

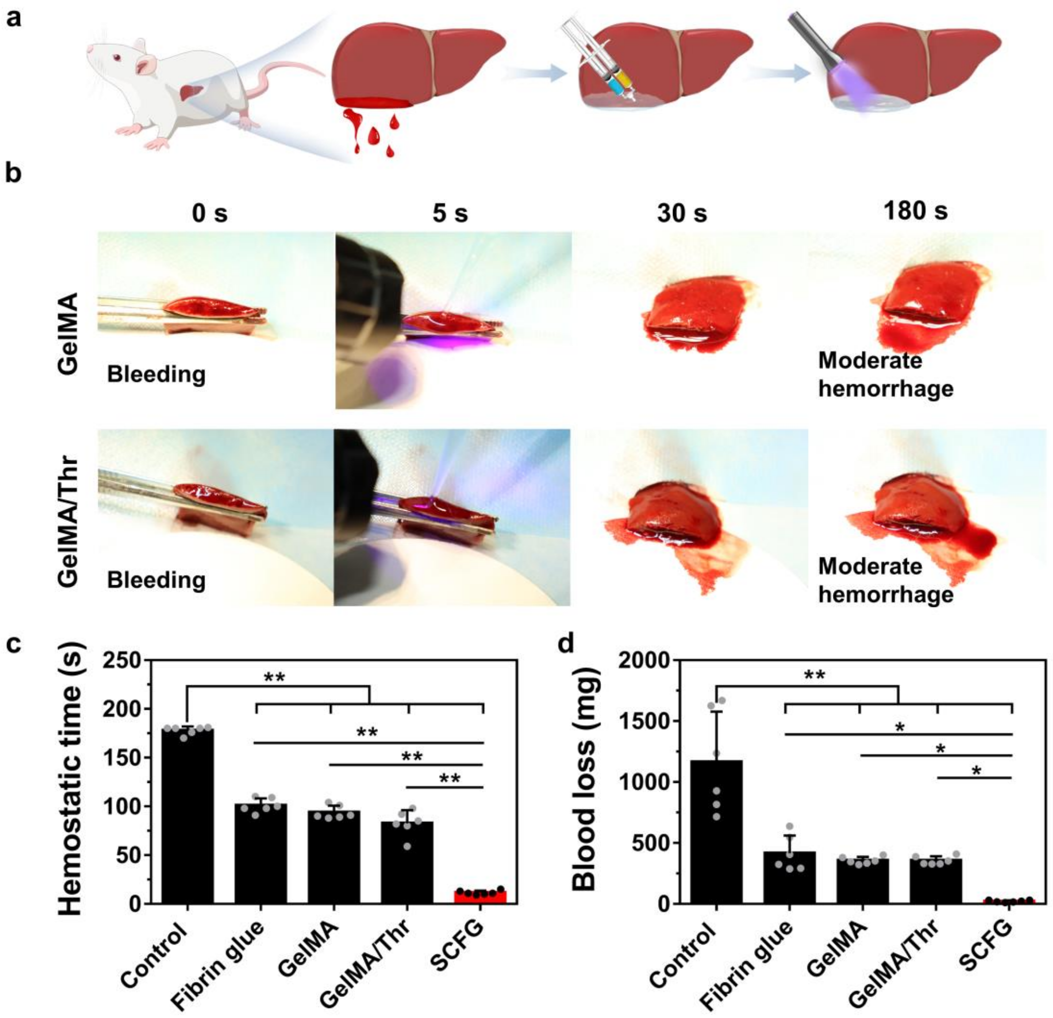

Figure S17 In vivo hemostatic sealing of a rat liver. a, Schematic illustration of liver bleeding in rat model. A part of the liver lobe was removed (length: 3 cm, width: 0.5 cm) to establish massive bleeding model. **b**, Experimental images of hemostatic treatment of liver defect in the GelMA and GelMA/Thr groups. **(c)** Hemostatic time and (**d**) blood loss of rat severe liver injury (n = 6). P values are determined by one-way ANOVA followed by the Tukey's comparison test. Error bars, mean ± SD. *$p < 0.05$; **$p < 0.01$.

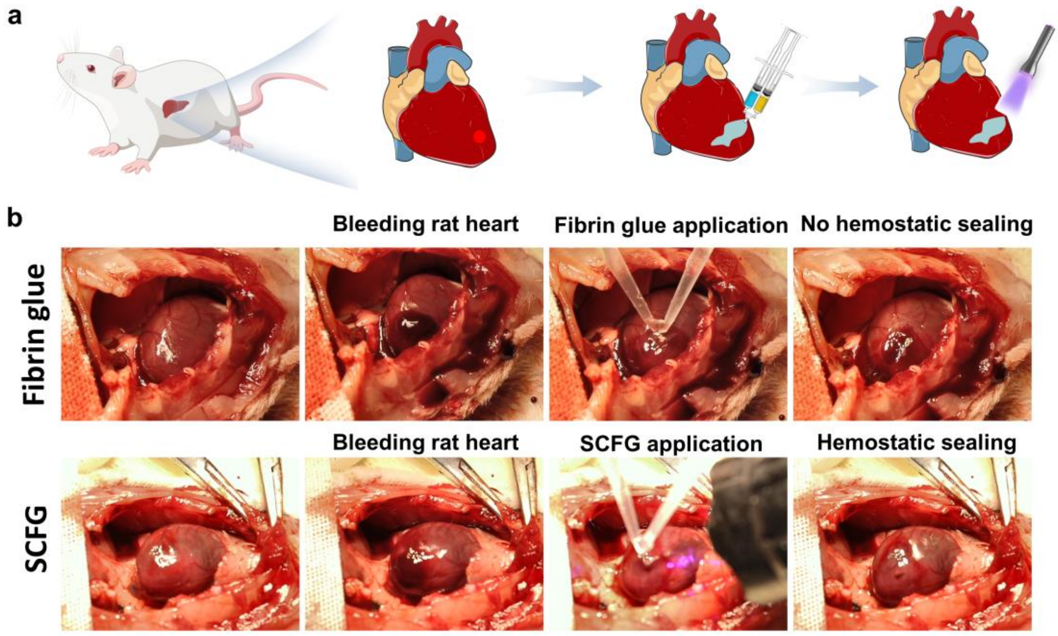

Figure S18 In vivo hemostatic sealing of a rat heart. a, Schematic illustration of a rat heart bleeding model. A cardiac perforation model (a 2.4 mm diameter ventricular wall injury) was made on rat heart, which resulted in an immediately high-pressure blood expulsion. **b,** Experimental images of hemostatic treatment in the fibrin glue and SCFG groups. Fibrin glue was injected immediately to cover the puncture site; however, the blood continued to hemorrhage from the ventricle because of weak adhesion to the surface of the bleeding heart. In contrast, SCFG rapidly formed a hemostatic seal on the ventricular injury within 15 s, and the puncture wound was tightly sealed to prevent high-pressure blood expulsion. This result demonstrated the excellent hemostatic capability of SCFG in acute hemorrhages.

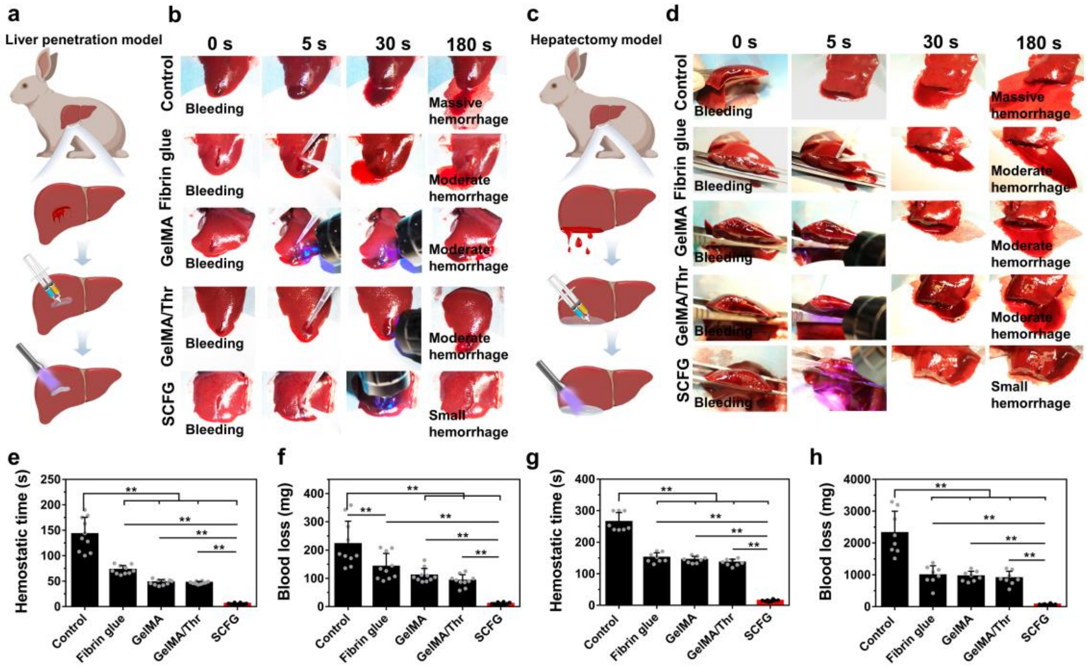

Figure S19 In vivo hemostatic sealing of two rabbit liver models. a, Schematic illustration of the rabbit liver penetration model. A liver incision (length: 10 mm, depth: 5 mm) was made to establish the liver penetration model. **b,** Experimental images of hemostatic treatment in the control, fibrin glue, GelMA, GelMA/Thr and SCFG groups. **c,** Schematic illustration of the rabbit hepatectomy model. A part of the liver lobe was removed (length: 3 cm, width: 0.5 cm) to establish the hepatectomy model. **d,** Experimental images of hemostatic treatment in the control, fibrin glue, GelMA, GelMA/Thr and SCFG groups. **(e)** Hemostatic time and (**f**) blood loss in rabbit liver penetration model (n = 10). **(g)** Hemostatic time and (**h**) blood loss in rabbit hepatectomy model (n = 8). P values are determined by one-way ANOVA followed by the Tukey's comparison test. Error bars, mean ± SD. *p < 0.05; **p < 0.01. SCFG presented significant hemostatic sealing capability in both the rabbit liver penetration model and the hepatectomy model in terms of sealing efficiency within 20 s.

Table. S1 Comparison of commercially available hemostatic material

Product Name	Product type	Major Components	Advantages	Disadvantages
Fibrin glue	Sealant	Fibrinogen and thrombin	— Biocompatibility<br>— Biodegradability<br>— Fast gelation	— Risk of transferring bloodborne disease<br>— Low adhesion<br>— High cost
Cyanoacrylate	Sealant	Cyanoacrylate monomer	— Fast polymerization<br>— High adhesion<br>— Low cost	— Toxic monomer<br>— Exothermic polymerization<br>— High rigidity<br>— Prolonged degradation
Surgiflo	Hemostatic matrix	Gelatin	— Providing tamponade effect<br>— Low immunogenicity	— Compression-related side effects<br>— Low efficiency for acute hemorrhage
Gelation sponge	Hemostatic sponge	Gelatin	— Biodegradability<br>— Low immunogenicity	— Compression-related side effects<br>— Low efficiency for acute hemorrhage
Surgicel	Hemostatic gauze	Cellulose	— Bactericidal property<br>— Biocompatibility	— Low efficiency for acute hemorrhage
Quikclot<br>(Combat Gauze)	Hemostatic gauze	Kaolin	— Procoagulant<br>— Low cost<br>— Good storability	— Exothermic reaction<br>— Inadaptation for internal hemostasis
Hemcon<br>(ChitoGauze)	Hemostatic gauze	Chitosan	— Various hemostatic mechanisms<br>— Antimicrobial property	— Inadaptation for internal hemostasis
Celox	Hemostatic gauze	Chitosan	— Various hemostatic mechanisms<br>— Antimicrobial property	— Inadaptation for internal hemostasis

Table. S2 Mechanical performance and hemostatic property of fibrin network or GelMA based adhesives in literature

Adhesives	Precursors	First network	Second network	Gelation time (s)	Storage modulus (Pa)	Injury model	Hemostatic time
GelMA/HA-NB[4]	GelMA solution; HA-NB solution;	GelMA	GelMA-HA (Schiff's bases)	1.38	3183	Rabbit severe liver injury model	in a few seconds
GelMA/Zinc ferrite/ silicate nanoplatelet[5]	GelMA/Zinc ferrite/ silicate nanoplatelet solution	GelMA	none	< 60	> 3000	Rat liver bleeding model	none
GelMA/hemocoagulase[6]	GelMA/hemocoagulase solution	GelMA	none	30	none	Rat liver injury model	45 s
tGNPsCH-FB[7]	Fg/chitin/gelatin nanoparticle solution; Thr/chitin/gelatin nanoparticle solution	Fibrin network	none	40	5460	Rat Liver Injury	89
Our work	GelMA/Fg solution; GelMA/Thr solution;	Fibrin network	GelMA	1.3	2790	Rat liver injury model	within 15 s

References

[1] C. Ghobril, M. W. Grinstaff, *Chem. Soc. Rev.* **2015**, *44*, 1820.

[2] G. Bao, Q. Gao, M. Cau, N. Ali-Mohamad, M. Strong, S. Jiang, Z. Yang, A. Valiei, Z. Ma, M. Amabili, Z. H. Gao, L. Mongeau, C. Kastrup, J. Li, *Nat. Commun.* **2022**, *13*, 5035.

[3] H. Yuk, J. Wu, T. L. Sarrafian, X. Mao, C. E. Varela, E. T. Roche, L. G. Griffiths, C. S. Nabzdyk, and X. Zhao, *Nat. Biomed. Eng.* **2021**, *5*, 1131.

[4] Y. Hong, F. Zhou, Y. Hua, X. Zhang, C. Ni, D. Pan, Y. Zhang, D. Jiang, L. Yang, Q. Lin, Y. Zou, D. Yu, D. E. Arnot, X. Zou, L. Zhu, S. Zhang, and H. Ouyang, *Nat. Commun.* **2019**, *10*, 2060.

[5] R. Haghniaz, H. Montazerian, A. Rabbani, A. Baidya, B. Usui, Y. Zhu, M. Tavafoghi, F. Wahid, H. J. Kim, A. Sheikhi, and A. Khademhosseini, *Adv. Healthcare Mater.* **2023**, e2301551.

[6] Y. Guo, Y. Wang, X. Zhao, X. Li, Q. Wang, W. Zhong, K. Mequanint, R. Zhan, M. Xing, and G. Luo, Sci. Adv. **2021**, *7*, eabf9635.

[7] M. N. Sundaram, V. Krishnamoorthi Kaliannagounder, V. Selvaprithiviraj, M. K. Suresh, R. Biswas, A. K. Vasudevan, P. K. Varma, and R. Jayakumar, *ACS Sustainable Chem. Eng.* **2018**, *6,* 7826.
